# DNA methylation predicts the outcome of COVID-19 patients with acute respiratory distress syndrome

**DOI:** 10.1186/s12967-022-03737-5

**Published:** 2022-11-12

**Authors:** Martina Bradic, Sarah Taleb, Binitha Thomas, Omar Chidiac, Amal Robay, Nessiya Hassan, Joel Malek, Ali Ait Hssain, Charbel Abi Khalil

**Affiliations:** 1 Department of Genetic Medicine, Weill Cornell Medicine, New York, USA; 2 Marie-Josee and Henry R. Kravis Center for Molecular Oncology, Memorial Sloan Kettering Cancer Center, New York, NY USA; 3 Division of Genomics and Translational Biomedicine, College of Health and Life Sciences- HBKU, Doha, Qatar; 4 Epigenetics Cardiovascular Lab, Weill Cornell Medicine-Qatar, Doha, Qatar; 5 Medical Intensive Care Unit, Hamad Medical Corporation., Doha, Qatar; 6 Nursery and midwifery research department, Hamad Medical Corporation., Doha, Qatar; 7 Genomics Core. Weill Cornell Medicine-Qatar., Doha, Qatar; 8 Joan and Sanford I. Weill Department of Medicine., Weill Cornell Medicine, New York, USA

**Keywords:** COVID-19, ARDS, DNA methylation, Epigenetics, Mortality, Survival, Biomarkers

## Abstract

**Background:**

COVID-19 infections could be complicated by acute respiratory distress syndrome (ARDS), increasing mortality risk. We sought to assess the methylome of peripheral blood mononuclear cells in COVID-19 with ARDS.

**Methods:**

We recruited 100 COVID-19 patients with ARDS under mechanical ventilation and 33 non-COVID-19 controls between April and July 2020. COVID-19 patients were followed at four time points for 60 days. DNA methylation and immune cell populations were measured at each time point. A multivariate cox proportional risk regression analysis was conducted to identify predictive signatures according to survival.

**Results:**

The comparison of COVID-19 to controls at inclusion revealed the presence of a 14.4% difference in promoter-associated CpGs in genes that control immune-related pathways such as interferon-gamma and interferon-alpha responses. On day 60, 24% of patients died. The inter-comparison of baseline DNA methylation to the last recorded time point in both COVID-19 groups or the intra-comparison between inclusion and the end of follow-up in every group showed that most changes occurred as the disease progressed, mainly in the AIM gene, which is associated with an intensified immune response in those who recovered. The multivariate Cox proportional risk regression analysis showed that higher methylation of the “Apoptotic execution Pathway” genes (ROC1, ZNF789, and H1F0) at inclusion increases mortality risk by over twofold.

**Conclusion:**

We observed an epigenetic signature of immune-related genes in COVID-19 patients with ARDS. Further, Hypermethylation of the apoptotic execution pathway genes predicts the outcome.

*Trial registration*: IMRPOVIE study, NCT04473131.

**Supplementary Information:**

The online version contains supplementary material available at 10.1186/s12967-022-03737-5.

## Background

COVID-19 is a novel coronavirus first discovered in Wuhan, China, in late 2019 and declared a pandemic by the World Health Organization (WHO) in March 2020 [1]. Two years later, several variants were detected, and over 5 million deaths were recorded [2].

Epigenetics refers to the study of gene activity regulation and expression changes that are not dependent on the DNA sequence [3]. DNA methylation, one of the hallmarks of epigenetics, involves the covalent addition of a methyl group to the 5′-carbon of a cytosine ring. Methylation is inversely correlated with gene expression [4]. For instance, hypermethylation is often associated with the downregulation of genes, recently demonstrated in the ACE2 gene [5]. Basic embryological and early developmental processes are controlled by DNA methylation in mammalians [6]. Further, DNA methylation is also involved in disease and upon exposure to environmental factors [7].

Patients with severe COVID-19 infection often suffer from respiratory failure and may require mechanical ventilation, associated with a mortality rate of up to 50% [8, 9]. Several predictors of outcomes in critically ill patients have already been identified. They include primarily clinical variables, biochemical markers, and comorbidities [10]. DNA methylation of host cells can be altered during infections, which modulates the immune response [11]. It has been recently shown that DNA methylation regulates the activity of the immune system in COVID-19 infections and is associated with clinical outcomes, such as the severity of the disease, its association with respiratory failure, and ICU admission [12–14]. However, data regarding death or recovery in COVID-19 patients is lacking. In this study, we report the presence of immune-related differentially methylated genes that predict survival in critically ill COVID-19 patients.

## Methods

### Participants

As part of the “Immune Profiling of COVID19-patients Admitted to ICU study (IMPROVISE) (clinicaltrial.gov identifier NCT04473131, start date 27th of April 2020), we recruited consecutively 100 critically ill COVID-19 patients with ARDS under mechanical ventilation (WHO clinical progression scale 7–9 [15]) at the intensive care unit (ICU) and 33 non-COVID participants from the blood donor unit at Hamad Medical Corporation (HMC), from April to July 2020. Detailed inclusion and exclusion criteria of participants are included in the Appendix. COVID-19 patients were included in the study upon their admission to the ICU (T1), then followed at four time points (T): day 7 (T2), day 14 (T3), day 21 (T4), and day 60 (post-T4), which is the recommended measure of patient survival according to the WHO Working Group on the common outcome measure set for COVID-19 clinical research [15]. After inclusion at T1, patients would progressively move to the next time point unless they die or recover, in which case their follow-up ceases. Recovery was defined as meeting the WHO clinical criteria of less or equal to 5, discontinuing mechanical ventilation, and discharge from the ICU to the COVID-19 ward. Blood samples were collected for epigenetic analysis at each time point.

### PBMCs isolation and DNA extraction

Seventeen ml of EDTA-coated blood was withdrawn from each participant. Peripheral blood mononuclear cells (PBMCs) were isolated by density gradient centrifugation using Ficoll-Paque Premium (GE Healthcare, Sweden) and SepMate tubes (STEMCELL technologies, USA). DNA from PBMCs was extracted (Allprep DNA/RNA mini kit, Qiagen, Germany) and then sequenced at the genomics core at WCM-Q.

### EPIC850 methylation quality control, data filtering, and normalization

To determine the DNA methylation status of study participants, we used Infinium MethylationEPIC 850 Array (~ 850,000 CpG sites) and its associated manifest file IlluminaHumanMethylationEPICanno.ilm10b4.hg19 with CpG sites annotation [16, 17]. Two hundred eighty-eight samples were collected: 100 from COVID-19 patients and 33 from controls at inclusion, and 155 for COVID-19 patients at different time points. We obtained DNA methylation beta values from the raw Intensity Data (IDAT) files using the minfi package in R 3.6.3 [18]. We then performed quality control (QC) by first calculating mean detection p-values across all samples and probes to identify failed samples and probes. All 288 samples were kept in the analysis. Thirty-one thousand seven hundred seventy bad-quality probes were removed. We then applied multiple filtering steps, including the removal of probes with SNPs using dropLociWithSnps function from minfi package [18] (26958 removed), cross-reactive probes were removed using xreactive_probes in package maxprobes (40148 removed) [19, 20], and finally probes on X and Y chromosomes (16109 removed). The final analysis set contained 288 samples and 750874 probes. After data filtering, we performed normalization using the preprocessQuantile function in the minfi package [18]. We then used the prcomp function to perform the principal component analysis (PCA).

### Estimation of the immune cells’ populations

FlowSorted Blood EPIC package in R [21] was used to estimate cell-type composition from normalized methylation data, including T lymphocytes (CD4 + and CD8 +), B cells (CD19 +), and monocytes (CD14 +), NK cells (CD56 +), and neutrophils. An accurate model to determine differences between immune cell proportions was determined based on the Akaike information criterion (AIC) and p-value using lm and glance functions in broom package (https://github.com/tidymodels/broom) in R. Differences between groups COVID-19/controls or recovery/death were then determined for each comparison using the most appropriate model among several tested ones that include immune cell proportions and clinical covariates that are statistically significant among studied groups.

### Methylation analysis and group comparisons

The getM function in the minfi package was used to convert row beta values to log-transformed M values used in downstream analysis. To estimate unknown variation within methylation data, we used the singular value approximation method from the sva package in R. We did not detect any novel variation in our sample [22]. We then used known covariates from the data and constructed different linear models to identify differentially methylated CpGs between studied groups. Models were then compared using the limma package function in R and AIC statistics. The model with the highest number of CpGs and the lowest AIC was selected as the best and used in the subsequent analysis. Multiple testing corrections and false discovery rates were calculated using the Benjamini–Hochberg procedure [23]. We also performed differentially methylated regions (DMRs) analysis in which we considered regions with five or more CpGs using the appropriate model established for CpGs, and the DMRcate package (https://bioconductor.org/packages/release/bioc/html/DMRcate.html) in R. Pathway enrichment analysis for all significant differentially methylated regions for all comparisons in this study was performed using 50 Human Hallmark pathways from MSigDB database [24], and *pathEnrich* function from splineTimeR R [25].

We first assessed methylation changes between COVID-19 patients and controls at the study’s inclusion, then looked at the differences between COVID-19 patients who recovered and those who died. Three different methods were applied for the latter. First, we looked for differences between both COVID-19 groups by comparing the methylation profile at inclusion to the last recorded methylation profile before “death” or “recovery.” Further, we compared COVID-19 patients who died to those who recovered at their baseline level than at discharge. Finally, we assessed differential methylation over time by testing the time-course differences between death and recovery. Since all comparisons were among the same samples at different time points, we only used the biological replicate as a covariate. To do this, we used the *splineDiffExprs* function in the splineTimeR package in R [25]. *SplineDiffExprs* fits the splines function for each phenotypic group across time points and replicates and compares their coefficient values. This allows us to detect differences over four time points for patients who recovered and those who died for immune cell proportions and methylation changes. The splinePlot function from the splineTimeR package was used to visualize the time-dependent behavior of CpGs in two phenotypic groups.

### Prediction of the outcome

The univariate Cox proportional hazard analysis of the CpGs was performed to identify methylation sites relevant to patient survival. We considered only differentially methylated CpGs between patients who recovered and those who died using the abovementioned three comparisons. Analysis was performed using all samples at inclusion. We first transformed methylation data to the standard normal distribution using Z-score. Then Cox survival model was fitted independently for each gene using RegParallel R package [26] using hospital stay as a time variable. The significance was determined using a log-rank test, and CpGs with p < 0.001 were considered significant. The patients were divided into high- or low-methylation groups using the median methylation Z-score as the cutoff point. Subsequently, the Kaplan–Meier (K-M) analysis was used to estimate the differences in survival between the two groups for the genes significant by the cox proportional hazardous model using the survival package in R (https://github.com/therneau/survival). Finally, we performed a Receiver Operating Characteristic (ROC) analysis of a logistic regression model where methylation was used to predict survival using the pROC package [27]. The area under the ROC curve (AUC) was calculated to compare the sensitivity and specificity of survival prediction.

## Results

### Baseline characteristics of the participants at admission

**Fig. 1:**
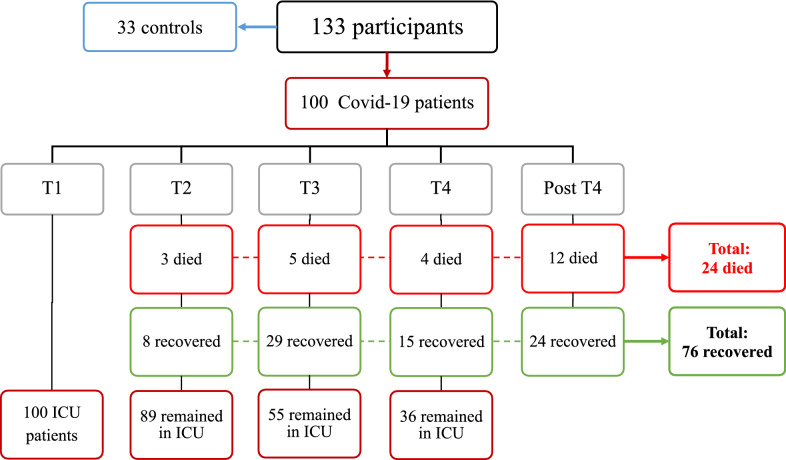
Flow chart of the study

One hundred thirty-three participants were included in the study, 100 COVID-19 participants and 33 volunteers at T1 (Fig. 1). COVID-19 patients were significantly older than controls and more likely to be South Asians (Table 1); hence, ethnicity and age were tested in the model used to compare both groups. Forty-one % of COVID-19 patients had diabetes, and 43% had hypertension. Nosocomial infections occurred in 55% of patients, and 30% received convalescent plasma therapy.

**Table 1:** Baseline characteristics of COVID-19 patients and controls

Variable	COVID-19 N = 100^a^	Controls N = 33^a^	p-valu^b^
Age	49 (42, 59)	40 (35, 45)	< 0.001
Gender (male)	95 (95%)	33 (100%)	0.3
BMI (kg/m^2^)	27.2 (24.6, 31.0)	29.3 (26.4, 31.2)	0.12
Ethnicity			
East Africa	0 (0%)	1 (3.0%)	< 0.001
Middle East	5 (5.0%)	11 (33%)	
North Africa	1 (1.0%)	0 (0%)	
Northeast Africa	5 (5.0%)	4 (12%)	
South Asia	78 (78%)	15 (45%)	
Southeast Asia	9 (9.0%)	2 (6.1%)	
Western Asia	2 (2.0%)	0 (0%)	
Duration of MV (days)	8 (4, 19)	–	
ICU LoS (days)	15 (10, 27)	–	
Hospital LoS (days)	27 (20, 44)	–	
ECMO	12 (12%)	–	
Nosocomial infections	55 (55%)	–	
Convalescent plasma therapy	30 (30%)	–	
*Diabetes status*			
Non diabetes	55 (55%)	–	–
Pre-diabetes	4 (4.0%)	–	
Diabetes	41 (41%)	–	
Hypertension	43 (43%)	–	–
Coronary artery disease	6 (6.0%)	–	–
Chronic kidney failure	11 (11%)	–	–
Chronic heart failure	2 (2.0%)	–	–

Data are represented as numbers (%) per each category for categorical variables and as median (IQR) for continuous variables

*ECMO* extracorporeal membrane oxygenation, *LoS* length of stay, *MV* mechanical ventilation

^a^Median (IQR); n (%)

^b^Wilcoxon rank-sum test; Fisher's exact test; Pearson's Chi-squared test

P-values were calculated with Fisher exact test or Wilcoxon rank-sum test

### Methylation differences between COVID-19 patients and controls at admission

We first performed PCA analysis to determine if population stratification is present in our dataset. Our principal component 1 (PC1) explained 13.5% of the variation. In comparison, PCA 2 explained 9.3% of the variation, and no population structuring was observed in relation to COVID-19 or control participants or ethnicity (Additional file 11: Figure S1, only COVID-19/controls PCA is shown).

**Fig. 2:**
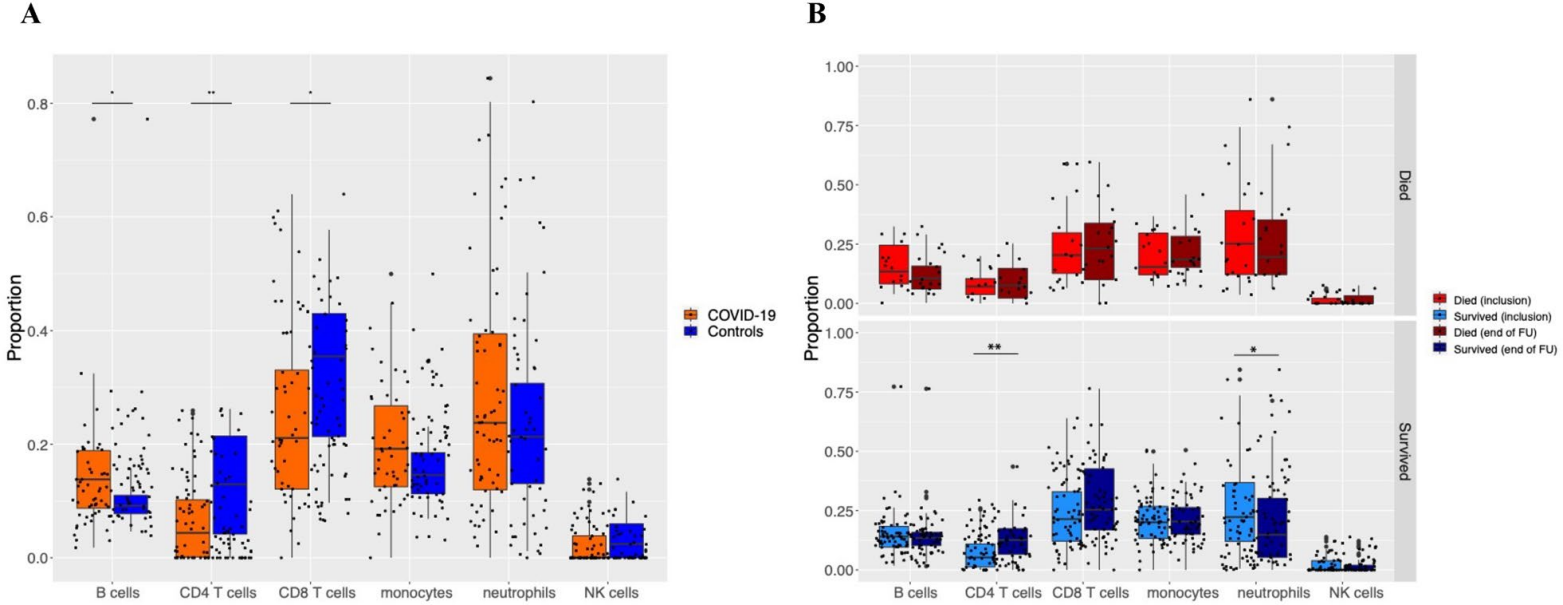
**A** The proportion of immune cells detected in controls and COVID-19 participants. The X-axis represents different cell types. The Y-axis represents the proportion of cell types derived from the deconvolution methods. The orange color represents COVID-19-patients, while the blue color represents controls. **B**. The proportion of immune cells detected at baseline and the final time point. Baseline-recovered, Baseline-died, recovered, and died are four different categories by which samples were grouped and compared for immune cell proportion

The proportion of immune cells is frequently variable in COVID-19 [28]; thus, we performed the deconvolution method and estimated the proportion of immune cells in every sample based on filtered and normalized data. Using a linear model, we then looked for cell proportions that significantly differed between groups. The best model (model 4: cell proportions ~ COVID-19/controls without the covariates) based on AIC was used (Additional file 1: Table S1). CD8 T cells, CD4 T cells, and B cells significantly differed between COVID-19 patients and controls: CD8 and CD4 T cells were significantly lower (FDR p < 0.05), and B cells were significantly higher (FDR p < 0.05) in COVID-19 patients compared to controls (Fig. 2A). These observations are consistent with previous observations [29].

**Fig. 3:**
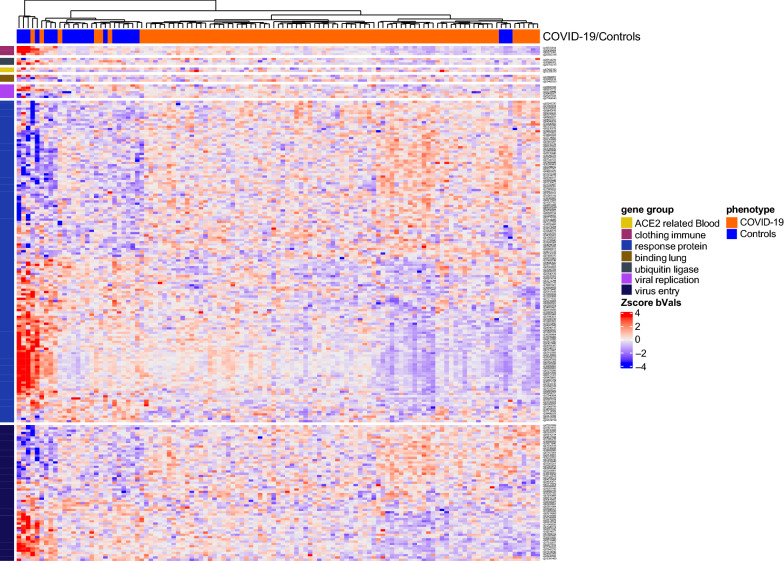
Differentially methylated CpGs between COVID-19 patients and controls. Heatmap represents significant changes in CpGs from 36 out of 40 genes previously associated with COVID-19^(13, 30–35)^. Heatmap represents methylation beta values (b-values) which were Z-score transformed (CD8 T, CD4 T, and B cell covariates were removed for visualization purposes.). Euclidean clustering distance and Ward.D2 clustering methods were used. Details on those genes and CpGs are shown in Additional file 2: Table S2C and D

To examine if changes in CpG methylation levels were associated with COVID-19 infection, we first established the best linear model for our analysis by testing the clinical covariates and the immune cell proportions. A total of 11 different models were tested (Additional file 11: Figure S2A), and the three immune cell types (CD8 T cells, CD4 T cells, and B cells) proportions contributed to the most significant number of CpGs based on AIC criterium; thus, they were included in the final model for differential methylation analysis (model F: CpG methylation ~ COVID-19/controls + the proportion of CD8 T cells/CD4 T cells/B cells). We detected 33.3% differentially methylated CpGs in COVID-19 patients in comparison to controls (a total of 133335 out of 750874; 71527 hypomethylated and 61808 hypermethylated, FDR, p < 0.05) (Fig. 3, Additional file 11: Figure S3, Additional file 2: Table S2A and B**)**. Gene-associated differentially methylated CpGs represent 0.7% (1054/133335) of the sites, while promoter-associated CpGs were more abundant with 14.4% (19238/133335), indicating a potential role in gene regulation. Observed methylation changes were associated with 20822 unique genes.

**Fig. 4:**
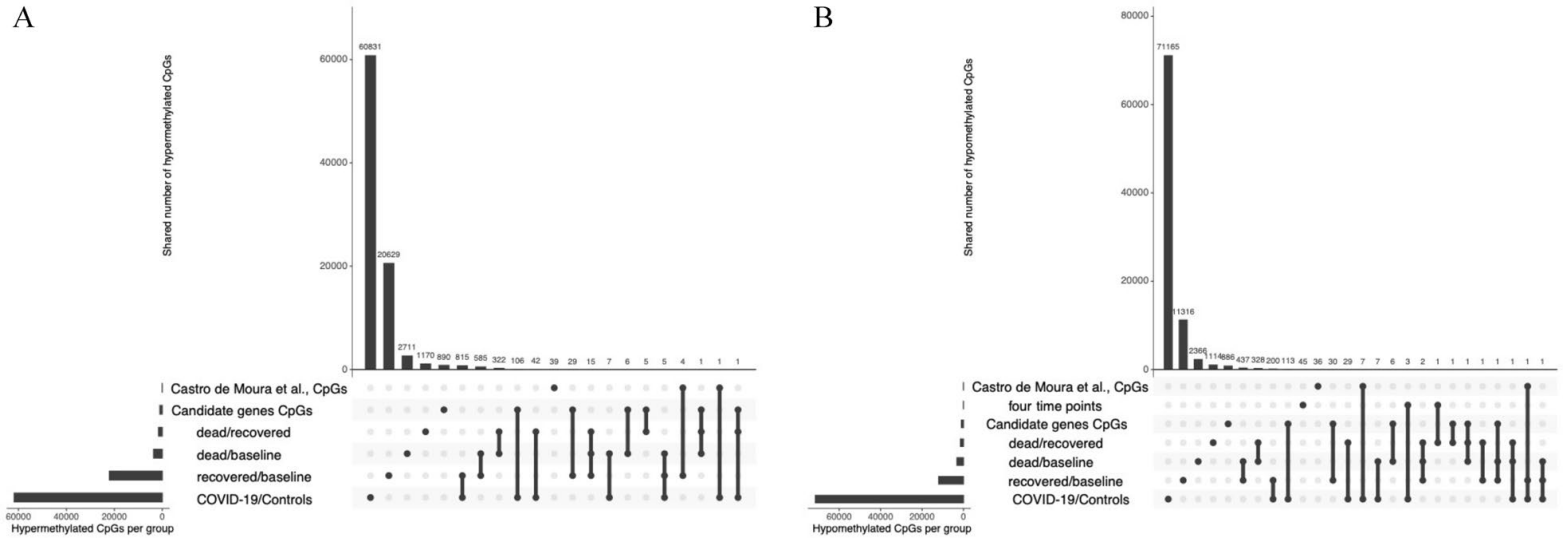
UpSet plot to summarize replication of our results from previous studies. UpSet panels summarize the differentially methylated CpGs that overlap our analysis and published COVID-19 studies. The bottom left horizontal bar graph labeled CpGs per group shows each panel’s total number of differentially methylated CpGs per group. The dots in each panel’s matrix represent unique and overlapping differentially methylated CpGs. Connected dots designate a particular intersection between different groups of CpGs, either by intersecting with published studies or within our research. The top bar graph in each panel recapitulates the number of differentially methylated CpGs for each unique or overlapping combination. **A**. Hypermethylated CpGs, **B**. Hypomethylated CpGs

To determine analysis reproducibility, we compared our observations with previously published data (Fig. 4). First, we investigated if 44 CpG sites that previously showed great accuracy in predicting COVID-19 severity [13] differed between COVID-19 and controls. Nine CpGs representing six genes including IFI44L (cg13452062), DDO (cg02872426), SGMS1 (cg10188795), CXCR2 (cg19225688), CCDC6 (cg04736673), CDC42BPB (cg02003183), cg06601098, cg11671940, and cg18523915 were also differentially methylated in our study. In addition, we have identified differentially methylated CpGs in the same genes, but not in the same sites as reported in the study from Castro de Moura et al. [13] and those included: two CpG in AIM2 and HLA-C genes, and one CpG site in each of the following genes: CELF4, CEP85L, KIFAP3, LCE1C, LHX6, MOBKL2A, PM20D1, PM20D1, SORCS1, UBAP2L, UBE2W, VIM, ZNF385D (Additional file 2: Table S2A and B).

Further, we examined if any previously reported COVID-19-associated genes had significant CpG methylation changes. Among 40 genes reported by Castro de Moura et al. [13] and replicated in other studies [30–35], we detected 220 CpGs from 39 genes in COVID-19 patients, 107 hyper-methylated and 113 hypomethylated (Figs. 3, 4). These included immune response, virus entry, viral replication, blood clotting, protein binding in lung cells, ubiquitin ligase, and ACE2-related genes (Additional file 2: Table S2C and D). We further performed pathway enrichment analysis to test the relationship between significantly methylated CpGs. We found significant enrichment of immune-related pathways, including interferon-gamma and interferon-alpha response, early estrogen response, apical surface, and UV response dn. These pathways were hypomethylated in COVID-19 patients, suggesting potentially induced expression of many immune-related genes. The mitotic spindle pathway was the only hypermethylated in COVID-19 patients (Table 2).

**Table 2:** Summary of differentially methylated pathways detected between COVID-19 patients and controls based on CpG sites

Hallmark pathway	N	DE	P.DE	FDR	Changes in CpGs (COVID-19 vs. controls)
Interferon gamma response	189	175	0.00	0.00	hypomethylation
Estrogen response early	189	174	0.00	0.02	hypomethylation
Apical surface	39	39	0.00	0.02	hypomethylation
Interferon alpha response	93	87	0.00	0.02	hypomethylation
Uv response dn	140	131.33	0.00	0.02	hypomethylation
Mitotic spindle	188	175	0.000	0.001	hypomethylation

Columns represent the following variables: *N* number of genes in the gene set, *DE* number of differentially methylated genes, *P.DE* p-value for over-representation of the gene set, *FDR* false discovery rate (p < 0.05)

We also performed a differentially methylated region (DMR) analysis using the same model as for CpGs, in which we identified 4788 hypermethylated in COVID-19 patients containing clusters of  ≥ 5 CpGs spanning 5723 genes (FDR p < 0.05; Additional file 3: Table S3). A total of 4347 hypo-methylated regions covering 5072 genes were also detected, indicating that DMRs spanned more than one gene. Pathway enrichment analysis was performed to determine relationships between genes detected by DMR analysis. We found significant enrichment of interferon-alpha response and Kras signaling pathways hypomethylated in COVID-19. In contrast, the mitotic spindle pathway was hyper-methylated, demonstrating similarity with individual CpG analysis (Table 3).

**Table 3:** Summary of pathways detected between COVID-19 patients and controls based on the differentially methylated region (DMR) analysis

Hallmark pathway	N	DE	P.DE	FDR	Changes in DMRs (COVID-19 vs controls)
Interferon alpha response	97	34	0.00	0.00	Hypomethylation
Kras signaling up	200	55	0.00	0.03	Hypomethylation
Mitotic spindle	199	68	0.00	0.00	Hypermethylation

Columns represent the following variables: *N* number of genes in the gene set. *DE* number of differentially methylated genes, *P.DE* p-value for over-representation of the gene set, *FDR* false discovery rate (p < 0.05). Analyzed regions are based on five or more CpGs

### Methylation changes between the dead and recovered COVID-19 patients

Three COVID-19 patients died at T2, five at T3, four at T4, and twelve at post-T4, representing 24 dead patients among the 100 initially included (24%) in 60 days. Patients who died were, on average, 11 years older than the ones who recovered (Table 4). As expected, they had more nosocomial infections and were more likely to receive extracorporeal membrane oxygenation (ECMO) (p < 0.05 for all). Interestingly, they did not suffer from more cardiovascular disease.

**Table 4:** Comparison between COVID-19 patients who survived *vs.* those who died

Variable	COVID-19, died N = 24^a^	COVID-19, survived N = 76^a^	p-valu^b^
Age	58 (52, 63)	47 (41, 56)	< 0.001
Gender (male)	24 (100%)	70 (92%)	0.023
BMI (kg/m^2^)	25.7 (24.5, 29.7)	27.5 (25.4, 32.1)	0.072
*Ethnicity*			
Middle East	1 (4.1%)	2 (2.6%)	0.14
North Africa	0 (0%)	1 (1.3%)	
Northeast Africa	2 (8.2%)	4 (5.2%)	
South Asia	18 (75%)	63 (82.8%)	
Southeast Asia	2 (8.2%)	5 (6.5%)	
Western Asia	1 (4.1%)	1 (1.3%)	
Duration of MV (days)	25 (19, 41)	8 (5, 18)	< 0.001
ICU LoS (days)	26 (20, 48)	15 (11, 29)	< 0.001
ECMO	7 (29%)	10 (13%)	0.001
Nosocomial infections	22 (92%)	42 (55.2%)	< 0.001
Convalescent plasma therapy	9 (37.5%)	25 (32.8%)	0.4
*Diabetes status*			
No diabetes	15 (62.5%)	41 (53.9%)	0.14
Pre-diabetes	1 (4.1%)	4 (5.2%)	
Diabetes	8 (33.4%)	31 (40.7%)	
Hypertension	11 (46%)	31 (40.7%)	0.5
Coronary artery disease	1 (4.1%)	4 (6.2%)	0.8
Chronic kidney failure	3 (12.5%)	8 (10.5%)	0.3
Chronic heart failure	1 (4.1%)	3 (3.9%)	0.9

Data are represented as numbers (%) per each category for categorical variables and as median (Interquartile range, IQR) for continuous variables

*ECMO* extracorporeal membrane oxygenation, *LoS* length of stay, *MV* mechanical ventilation

^a^Median (IQR); n (%)

^b^Wilcoxon rank-sum test; Fisher's exact test; Pearson's Chi-squared test

P-values were calculated with Fisher exact test or Wilcoxon rank-sum test

#### Inter-comparison of DNA methylation changes between baseline and the last recorded time point in COVID-19 groups

**Fig. 5:**
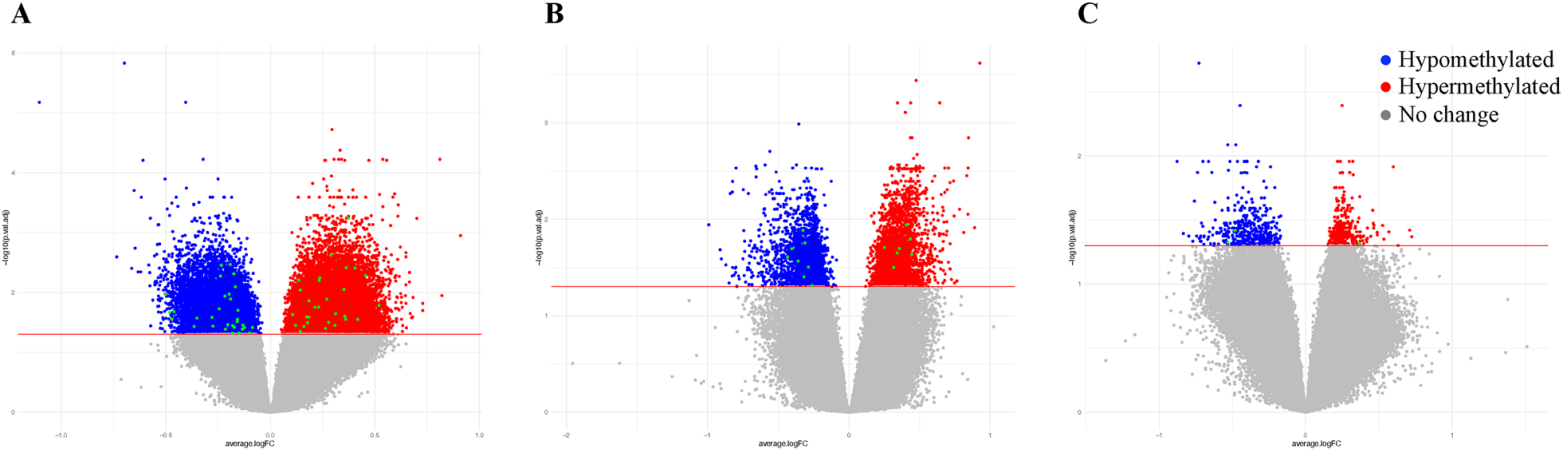
Differential methylation between dead and recovered COVID-19 patients. **A**. Volcano plot showing differences between COVID-19 recovered patients and their baseline. **B**. Volcano plot showing differences between COVID-19 dead patients and their baseline, **C**. Volcano plot showing differences between dead and recovered patients at their latest time point. Volcano plots in A, B, and C show differential CpGs methylation over 750874 CpG positions. The red line designates the genome-wide significance threshold of a Benjamini–Hochberg corrected p < 0.05. Green dots represent significantly different CpGs from the previously reported genes associated with COVID-19 [13, 30–35]. Red dots represent hypermethylated CpGs; blue plots represent hypomethylated CpGs. Grey dots represent non-significant CpGs

We compared the immune cell content and methylation profile at inclusion to the last recorded methylation profile before death or recovery. In recovery, we detected a higher proportion of CD4 T cells and a lower proportion of neutrophils in comparison to their baseline immune cell content (adjusted p < 0.05 for both) (Additional file 4: Table S4A, Fig. 2B). Further, we identified 11989 hypomethylated and 22082 hypermethylated CpGs (Fig. 5A, Additional file 4: Table S4B) entailing multiple pathways. Hypermethylated CpGs were enriched in the inflammatory response, interferon-alpha response, heme metabolism, TNF-alfa signaling via NF-kB, estrogen response early, Kras signaling up, uv response dn, il2 stat5 signaling, mitotic spindle, interferon-gamma response, il6 jak stat3 signaling, apical junction, and myogenesis pathway (Additional file 4: Table S4C). Hypomethylated CpGs were enriched in allograft rejection, mitotic spindle, and myc targets v1 pathways. In patients who died, the proportion of immune cells between baseline and the last recorded time point before death did not differ (Additional file 4: Table S4D**, **Fig. 3). Surprisingly, those patients expressed a smaller number of differential methylation changes than their baseline value compared to changes observed in those who recovered. We detected 3150 hypomethylated and 3652 hypermethylated CpGs (Fig. 5B, Additional file 4: Table S4E). There were no significant changes in pathways related to these methylation changes after the false discovery rate (FDR) correction. (Additional file 4: Table S4F).

We also performed a DMR analysis in which we considered regions with five or more CpGs. There were 310 hypermethylated regions relative to 363 genes and 82 hypomethylated regions relative to 102 genes in recovered patients (Additional file 4: Table S4G). There were no significant pathways after FDR correction. However, some of the same pathways related to immunity that were significant in individual CpGs analysis also showed nominal significance for DMR (Additional file 4: Table S4H). We also tested if any DMRs were significantly associated with death. We identified 35 regions encompassing 45 genes that were hypermethylated in patients who died. Only three regions were hypomethylated in the same comparison, and they spanned four genes (i.e., GNAS, MEST, RP1-309F20.3) (Additional file 4: Table S4I). Nevertheless, we did not detect significant pathways related to those DMR changes (Additional file 4: Table S4J).

#### Intra-comparison of DNA methylation differences between both groups at inclusion and the end of follow-up

We compared COVID-19 patients who died to those who recovered at their baseline level. We first tested the models, including different combinations of the clinical covariates, to determine the optimal model to compare immune cell proportion differences. The model without covariates (mod7; cell proportions ~ Dead/Recovered) was selected as the best model based on this criterium and was used in data analysis (Additional file 5: Table S5A). We did not identify significant differences between “dead” and “recovered” patients at baseline or last time point for immune cells (Additional file 5: Table S5B, C). We next established the best model for differential methylation comparisons of these groups by testing different covariates**.** The model “none” (without any covariates) fitted most of the CpGs based on both criteria; thus, this model was selected for subsequent analysis. (Additional file 11: Figure S2B).

CpG sites or regions suggest that the methylation changes at baseline (baseline died vs. baseline recovered) did not significantly affect the outcome. However, comparing the same patients at their last time point resulted in 1478 hypomethylated and 1557 hypermethylated CpGs in patients who died compared to those who recovered (Fig. 5C, Additional file 6: Table S6A). No significant pathways were detected for this comparison. Furthermore, we identified hypermethylated DMRs in 156 regions near 190 genes and 102 hypomethylated regions in 133 genes. (Additional file 6: Table S6B). There were no significant pathways based on genes in DMR pathways. We also did not find any immune cell proportion differences in this comparison.

**Fig. 6:**
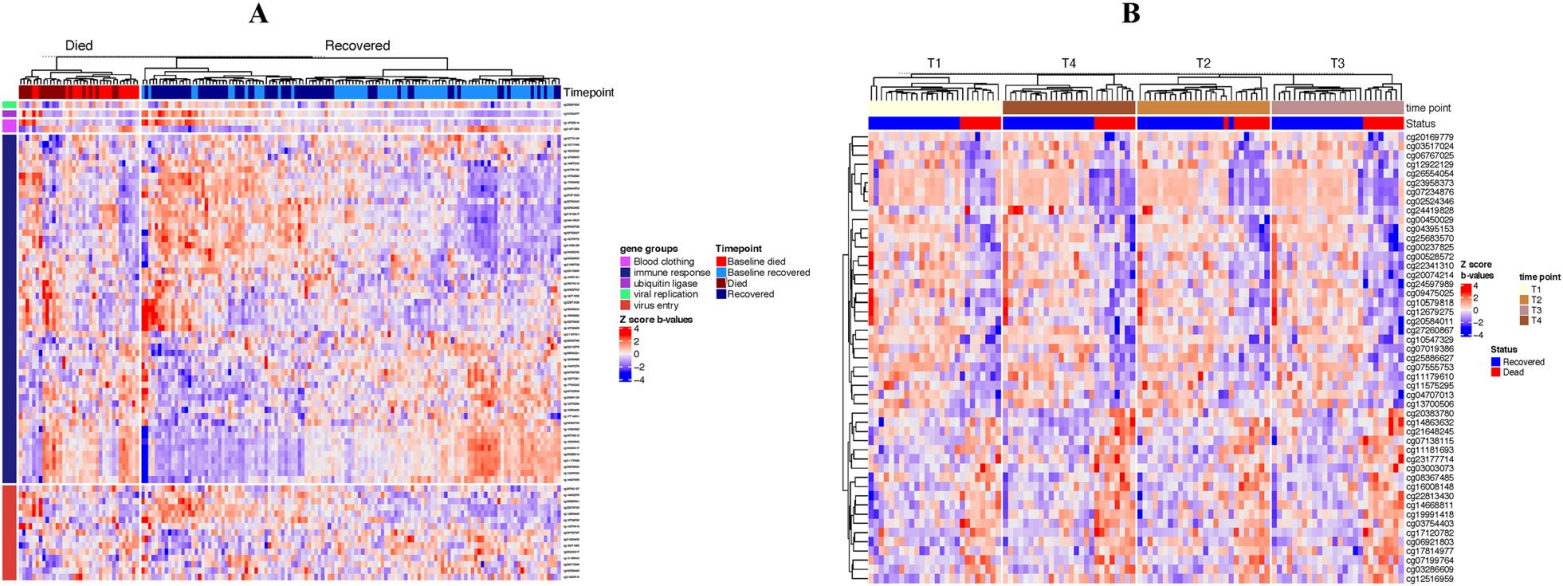
Heatmap representing significant changes in CpGs from genes previously associated with COVID-19 [13]. Heatmap represents methylation beta values which were Z-score transformed (the euclidean clustering distance and Ward.D2 clustering methods were used). Details on these genes and CpGs are shown in Additional file 4: Table S4

Compared to the published study of Castro de Moura et al. [13], which predicts 44 CpGs for severe COVID-19 cases, we have identified five CpGs that differ only between baseline and the final time points in patients who recovered. None differed between patients who died and their baseline (Fig. 4). Four CpGs (cg11671940; RP11-351M16.3, cg10188795; SGMS1, cg17515347, and cg24145401; AIM2) were hypermethylated in comparison to baseline, and one was hypomethylated (cg06601098), which did not belong to any gene region and was an open sea CpG. All but one of those CpGs was hypomethylated in severe COVID-19 cases in Castro de Moura et al. [13]. They were significantly hypermethylated in recovered patients, suggesting their potential to improve outcomes. The exception of that was cg06601098 which was hypomethylated in severe COVID-19 patients who recovered in Castro de Moura et al.’s study. [13]. However, none of these changes were significant when comparing the last time point in recovered vs. dead patients. Furthermore, we also identified changes in CpGs from 40 candidate genes from previous studies. Here we observed 29 hypermethylated and 31 hypomethylated CpGs in recovered at the last time point with respect to baseline (Figs. 6, 4). Patients who died had seven hypermethylated and eight hypomethylated CpGs. One of these CpGs (cg16371860 in TMPRSS2 gene) was significantly hypomethylated in both recovered and those who died; thus, it is probably not of great interest as a potential marker for recovery. Comparing recovered and died at the last time point with published data did not yield any significant CpGs from Castro de Moura et al. [13]. However, we found nine CpGs from 40 candidate genes, including two hypo-methylated CpGs in dead patients (promoter-associated OAS1; cg18217049, and TMPRSS2; cg19020860). We also saw three hypermethylated CpGs in deceased patients from STAT3 (cg17833746, cg24312520, cg24718015), one from OAS2 (cg19371652), one from LZTFL1 (cg09709426), and one that was from the TBK1 gene (cg13540592) (Figs. 6, 4). These CpGs were not enriched in any pathways.

#### Time course of differential methylation induced by critical COVID-19 illness

**Fig. 7:**
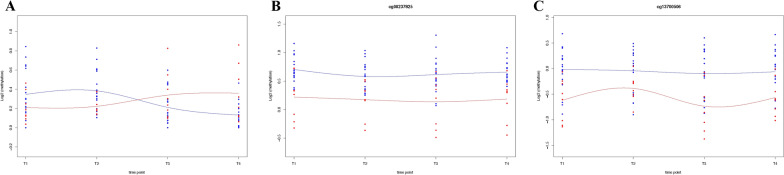
Summary of immune cells and methylation changes over four time points in COVID-19 patients. **A**. Spline regression plot of neutrophile changes over four time points in dead (red color) vs. recovered patients (blue color). **B**. Spline regression plot of significant CpGs (cg00237825) over four time points in recovered vs. dead patients for DEFB115 **C**. Spline regression plot of significant CpGs (cg13700506) over four time points in recovered vs. dead patients for DEFB116. Spline plots show the spline regression model fitted to the four time points neutrophile proportion data (**A**) and methylation (**B**). The blue line represents the fitted model for the recovered, while the red line represents dead patients. Blue and red dots represent the proportion of neutrophils/methylation of the biological replicates for dead and recovered patients. Vertical lines are the endpoints and interior knots representing 0.33 and 0.66 quantiles

To determine methylation changes over time, we analyzed patients who either died or recovered at day 60 (post T4) for all the four time points (patients who did not reach T4 were excluded from this analysis. First, we looked at whether patients who died and recovered differed in immune cell dynamics. Immune cell proportion analysis using spline function identified significant changes in neutrophils (adjusted p < 0.05). Neutrophils showed a sudden increase in T3 and T4 in patients who later died. In contrast, those who recovered showed the opposite scenario (Fig. 7. A, Additional file 7: Table S7A), suggesting the critical importance of these cells in the clinical outcome.

**Fig. 8:**
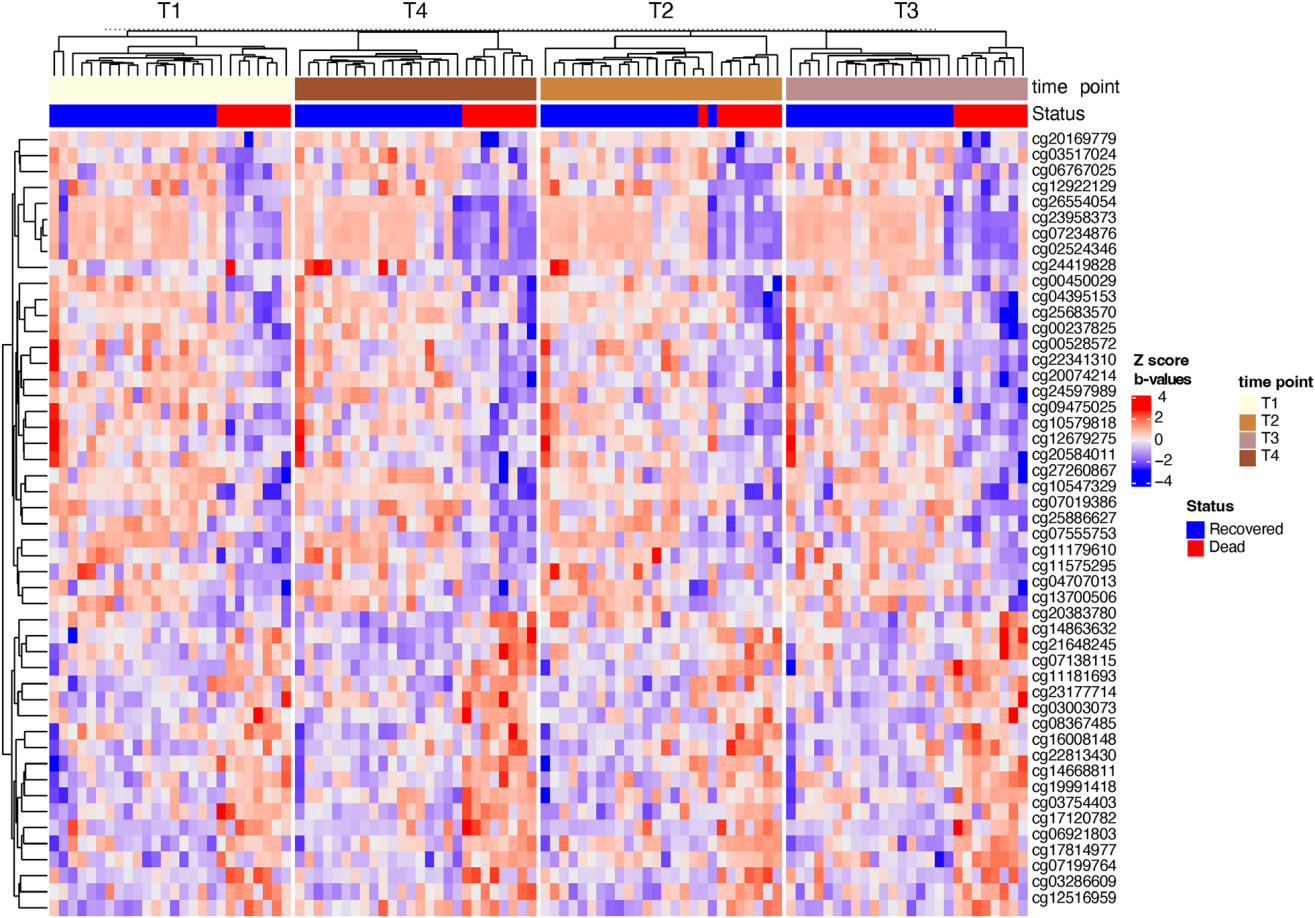
Heatmap representing significant changes in CpGs between patients who died and those who recovered over four time points. Heatmap represents methylation beta values which were Z-score transformed (the euclidean clustering distance and Ward.D2 clustering methods were used). Details on these CpGs are shown in Additional file 7: Table S7B

We identified differences in methylation trends between patients who died and those who recovered over four time points for 49 CpG sites that correspond to 27 genes (Fig. 8, Additional file 7: Table S7B), most of which are known to regulate the activity of protein binding Among those 27 genes, 19 were already reported in COVID-19 either in the viral entry and binding to receptors, or in clinical prediction such as the severity of the disease or its associations with end-organ damage (Additional file 8: Table S8).

Among genes not reported in COVID-19, two are known to play a role in the immune system: DEFB116 and DEFB115. Those genes belong to the beta-defensins system, which is a vital part of the innate immune response and plays an essential role in protection against infections [36]. We identified differential methylation of CpGs in those genes over all four time points (Fig. 7B and C). DEFB115 was overall less methylated in COVID-19 patients who died, suggesting a potentially increased expression in critical COVID-19 patients. DEF116 was only hypomethylated in patients who died.

### Prediction of the outcome

We tested all CpGs (a total of 40,956) issued from the inter-comparison between baseline and the last recorded time point before the outcome in both COVID-19 groups, the intra-comparison between both groups at inclusion and by the end of follow-up, and those issued from the time course of differential methylation. A total of 13 CpGs corresponding to 8 genes predicted the outcome. Three of those genes are issued from the comparison of methylation changes between baseline and the last time point in patients who survived (PSMB9, MFHAS1, and MRPS2), and five from the comparison of baseline and the last points in patients who died (MAT2B, YY1P2, ROCK1, ZNF789, H1F0).

**Fig. 9:**
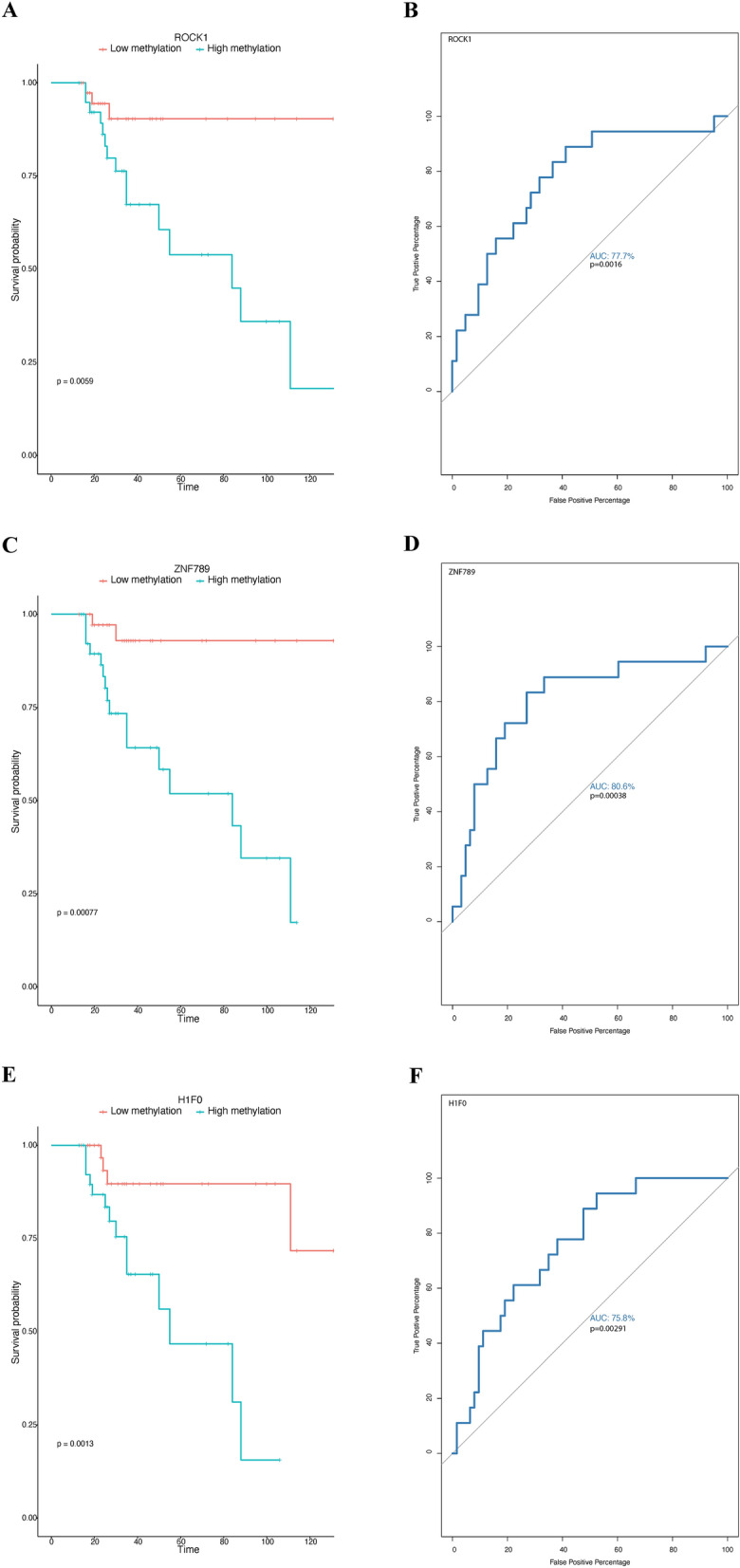
Kaplan–Meier and ROC analysis of genes that are top predictors of COVID-19 survival showing the three genes that increase mortality (**A** Kaplan–Meier plot represents the difference in survival probability between high and low methylation associated with CpG within a gene region. High and low methylation represents two groups determined based on the median of methylation Z-score as a cutoff. The X-axis represents time. The Y-axis represents survival probability. The tick marks indicate the censored patients. **B** ROC curves of the differentially methylated genes were used to demonstrate the sensitivity and specificity in predicting the survival of COVID-19 patients at inclusion. The X-axes show the false positive percentages, while the y-axes show the true positive percentages. P values on the plots represent the significance of logistic regression, where methylation was used as a dependent variable and survival (dead/alive) as an independent variable. The area under the curve (AUC) is shown for each gene showing how good the model is for hazard prediction

**Fig. 10:**
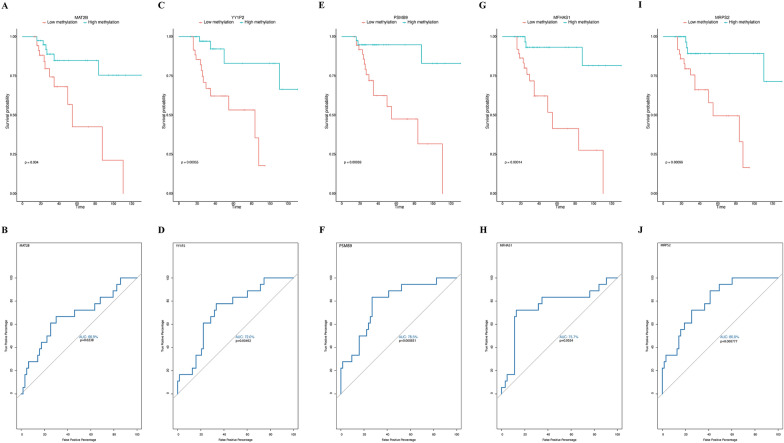
Kaplan–Meier and ROC analysis of genes that are top predictors of COVID-19 survival showing the five genes that decrease mortality) **A** Kaplan–Meier plot represents the difference in survival probability between high and low methylation associated with CpG within a gene region. High and low methylation represents two groups determined based on the median of methylation Z-score as a cutoff. The X-axis represents time. The Y-axis represents survival probability. The tick marks indicate the censored patients. **B** ROC curves of the differentially methylated genes were used to demonstrate the sensitivity and specificity in predicting the survival of COVID-19 patients at inclusion. The X-axes show the false positive percentages, while the y-axes show the true positive percentages. P values on the plots represent the significance of logistic regression, where methylation was used as a dependent variable and survival (dead/alive) as an independent variable. The area under the curve (AUC) is shown for each gene showing how good the model is for hazard prediction

Higher methylation of ROC1, ZNF789, and H1F0 increased the mortality risk (cox proportional HR = 2.43, 95% CI [1.58–3.6]; 2.29, 95% CI [1.49–3.53]; 2.62, 95%, CI [1.60–4.29]; respectively) (Fig. 9, Additional file 9: Table S9) whereas higher methylation of PSMB9, MFHAS1, MRPS2, MAT2B, and YY1P2 decreased it risk (cox proportional HR = 2.43, 95% CI [1.58–3.6]; 2.29, 95% CI [1.49–3.53]; 2.62, 95%, CI [1.60–4.29]; respectively) (Fig. 10). The ROC curves of sensitivity and specificity of the model showed a very good prediction of the outcome for ZNF789 (80.6%) and MRPS2 (80%), a good one for PSMB9 (78.5%), MFHAS (75.7%), H1F0 75.8%), YY1P2 (72%), and ROCK1(77.7%) genes, and a fair one for MAT2B (66.9%) (Figs. 9 and 10). Those genes are mostly linked to inflammation or DNA regulation. Interestingly, seven of those genes have been reported in previous COVID-19 studies (Additional file 10: Table S10). Further, we looked at the string database to assess protein interaction in those genes. At the same time, no interaction exists in the five genes that decrease mortality and enrichment in the Reactome pathway [37], known as the “Apoptotic execution Pathway,” exists [38].

## Discussion

To our knowledge, this is the first longitudinal study to investigate the methylation profile in critically ill COVID-19 patients with ARDS under mechanical ventilation and identify a methylome signature that predicts survival.

We showed that the epigenetic signature of critical COVID-19 infection is enriched for immune response pathways, particularly type I Interferon signaling, which is a key signature of the host response to this virus [14, 33, 39, 40]. Interferon-driven response plays a vital role in shaping the fate of a viral infection, affecting the activation and differentiation of immune cells and the virus spread [14, 33, 39, 40]. Other differentially methylated genes also contribute to immune-related functions and viral pathogenesis. For example, IFNAR1 and IFNAR2 genes partake in type I interferon-related pathways as main receptors for interferon-alpha and beta [41]. Another gene is CLEC4M which encodes for the CD209L receptor and mediates the virus entry to epithelial and endothelial cells of various tissues [42].

Compared to controls, critically ill COVID-19 patients showed a similar differential methylation pattern to previously reported studies [12, 13, 30–35]. Konigsberg et al. recently identified 13033 differentially methylated CpGs, from which we have confirmed 3613 that represent 2290 genes [40]. In particular, we found that the probes of robust predictors of COVID-19 were hypomethylated in our patients, including genes involved in interferon regulation and viral response. This may suggest an increased expression of those genes during critical COVID-19 infection, which has also been reported earlier [14].

Interestingly, at inclusion, we did not observe any intra-differences in DNA methylation between dead and recovered groups. However, the same comparison showed significant differences at the last recorded time point, suggesting that most changes occurred as the disease progressed. Further, the inter-comparison of methylation changes between baseline and the last recorded time point revealed hypermethylation of pathways linked to host immune response such as interferon-alpha, TNF alpha, IL-6, and IL-2 signaling in patients who recovered, but not in those who died. Among the reported genes in patients who recovered, AIM plays a vital role in the immune response. It initiates the inflammatory cytokines release upon sensing exogenous nucleic acid inside the host cell, followed by pyroptosis (lytic cell death) [43]. It has been associated with intensified immune responses to COVID-19 [44]. CpGs in that gene are promoter-associated, and their hypermethylation suggests reduced AIM expression in patients just before recovery; thus, its reduced activity might be related to improvement and survival. Among the genes we reported in patients who died, LZTFL1 is known to inhibit epithelial-mesenchymal transition (EMT) in the lungs in the presence of inflammation or cancer [45, 46]. EMT is a well-known pathway in fibrosis that is activated in prolonged lung inflammation and tissue injury [47]. In-vitro studies showed that COVID-19 upregulates EMT pathway genes [48]. In our study, LZTFL1 was hypermethylated, which would be translated by a decrease in its expression, less inhibition of EMT, and progressive lung injury.

Time course differential methylation analysis identified 49 CpGs, two of which are beta-defensin genes. Beta-defensins are antimicrobial peptides, modulators of microbiome diversity and host-microbe equilibrium in the mucosa of oropharyngeal and tracheal highways, and regulators of inflammatory responses secreted by neutrophils during infections [49]. They are one of the primary arms of the innate immune system, contributing to immune cell activation and proliferation [36]. In our study, the lower methylation of those genes in non-survivors suggests a higher expression of antimicrobial peptides throughout their ICU stay.

CD4 and CD8 T cells are critical elements in anti-viral immunity; they work harmoniously to recognize viral antigens, proliferate, kill infected cells, neutralize the virus, and memorize the viral print to respond faster in the case of future encounters [50]. Our deconvolution analysis confirmed a lymphopenic profile (low CD4 and CD8 proportion) in COVID-19 patients upon admission to the ICU. This is consistent with previous reports and could be interpreted as a sign of dysfunctional or exhausted immune cells [51]. At late stages, CD4 T cell proportion increased in survivors, indicating the restored function of the immune system [51]. Neutrophils showed a sudden increase in patients who died at the last two time points, which could reflect a prolonged inflammatory response, contributing to severe conditions [52]. One of the plausible theories behind the increase in neutrophils and hyper expression of the beta-defensins and other immune-related genes is the re-occurrence of a cytokine storm before death.

Among the genes that predict mortality, ROCK1 plays a crucial role in apoptosis by regulating membrane blebbing, a characteristic feature of apoptotic cells [53], and H1F0 through apoptosis-induced DNA fragmentation and cellular component disassembly [54]. It is well known that the apoptotic execution pathway initiates cell death once activated by an abnormal immune reaction [55]. This finding was reported in cancer cells that resist the activation of this pathway to escape anti-cancer therapeutics in vitro [56] but never reported in vivo in COVID-19 infections. On the other hand, higher methylation of PSMB9, MRPS2, MFHAS1, and MAT2B genes are known to be expressed in COVID-19 patients with high viral load or severe infection [57–61], which could translate to a lower expression of those genes. It might be possible that a less severe infection at ICU admission predicts better survival.

Cumulative data suggest that epigenetics play an important role in the pathophysiology of several pathologies such as cardiovascular disease, diabetes, and cancer [7, 62–63]. Recently, epigenetic markers were suggested as potential indicators and biomarkers for disease detection and progression [64]. In acute Charcot disease, a rare diabetes complication characterized by bone destruction, we have previously shown the presence of differentially methylated genes involved in the migration process during monocyte differentiation into osteoclasts [65]. Further, epigenetic-based therapy is increasingly used in several disciplines such as immunotherapy and cancer [66, 67]. Current experimental approaches in infectious diseases in general and viral infections, mainly, are promising [68, 69].

This study has a few limitations. The sample size of our patients was relatively moderate; hence a higher number of participants might have enabled us to detect more methylation calls knowing that power calculations for the sample size are not established for epigenetic analysis. We conducted the study in early 2020 during the first wave of COVID-19 when the Alpha variant was the only one universally reported. Therefore, we cannot ascertain that the same methylation changes exist with different variants in vaccination or the constant changes in drug therapies.

## Conclusion

In total, we identified an epigenetic signature in critically ill COVID-19 patients with ARDS that predicts the clinical outcome. While immune-related pathways, interferon-alpha and -gamma, were initially the main biological mechanisms differentiating critically ill COVID-19, an epigenetic signature set of eight genes predicted survival. Further studies are needed to elucidate the potential use of the methylome as a biomarker of the disease and, most importantly, to assess DNA methyltransferase, nucleoside inhibitors, or other pharmaceutical potential epigenetic-targeted therapies in COVID-19.

### Supplementary Information

**Additional file 1:Table S1.** Baseline characteristics of COVID-19 patients and controls.

**Additional file 2: Table S2.** Summary of different models tested for estimating differences between controls and COVID-19 patients for immune cell proportions. Adjusted R2, residual standard error (sigma), AIC, and p.value for each tested model for each cell type are shown. The following models were tested, mod1; Age and ethnicity as covariates, mod2; age as a covariate, mod3; ethnicity as a covariate, mod4; no covariates.

**Additional file 3: Table S3.** Summary of identified differentially methylated CpGs between COVID-19 patients and controls. A. All significant CpGs B. Variable description from Table S2A. C. Significant CpGs from genes previously described as COVID-19 important [1]. D. Functional annotation of genes from Supplemental table 3C.

**Additional file 4: Table S4. **Summary of differentially methylated pathways detected between COVID-19 patients and controls based on CpG sites.

**Additional file 5: Table S5.** Summary of differentially methylated CpGs in recovered and died COVID-19 patients. A. Immune cell comparison between baseline and recovered pairs, B. Significant CpGs between baseline and recovered pairs, C. CpG pathways between baseline and recovered pairs, D. Immune cell comparison between baseline and died pairs, E. Significant CpGs between baseline and died pairs, F. CpG pathways between baseline and died pairs.

**Additional file 6: Table S6.** Analysis of dead and recovered+A3 COVID-19 patients for immune cell proportions. A. Summary of different models tested for immune cell proportions. Adjusted R2, residual standard error (sigma), AIC, and p-value for each tested model for each cell type are shown. The following models were tested, mod1; Age + MV days + Gender + ICU LoS + ECMO + Nosocomial infections, mod2; Age + Gender + ICU LoS + ECMO + Nosocomial infections, mod3; Age + MV days + ICU LoS + ECMO + Nosocomial infections, mod4; Age + ECMO + Nosocomial infections, mod5; Age + Nosocomial infections, mod6; Age, mod7; no covariates. B. Immune cell differences between dead and recovered at baseline, C. Immune cell differences between dead and recovered at last time point.

**Additional file 7: Table S7.** Summary of immune cell changes and differentially methylated CpGs between recovered and dead patients over four time points 7A. Immune cell changes between recovered and dead patients over four time points, 7B. Differential methylation of CpGs between recovered and died patients over four time points. The b_0, b_1, b_2, and b_3 coefficients correspond to the reference model parameters, where survival phenotype is used as a reference. The d_0, d_1, d_2, d_3 coefficients represent the differences between the reference model and the model parameters in the compared group (died). AveExprs refers to the average log2-expression for an individual immune cell proportion or CpG. The F column contains moderate F-statistics, P-value -raw p-value, and adj.P.Valu- Benjamini-Hochberg adjusted p-value. Other column variables are described in Supplemental table 2B.

**Additional file 8: Table S8.** Description of 27 genes from 49 differentially methylated CpGs between survived and dead  patients over four time points. A summarized description of the 27 genes obtained from Supplementary Table 7B, collected from Gene Ontology (GO) to identify the functional annotation of each gene and recently published COVID-19-related articles to highlight the role of each gene in relation to COVID-19.

**Additional file 9: Table S9.** Summary of univariate Cox proportional hazard analysis of the previously identified CpGs. Column variables represent Name; chr; chromosome number, pos; position, CpG name, relation_to_Island; where is CpG located in relationship to island, UCSC RefGene Name; UCSC gene name, UCSC RefGene Accession; UCSC gene accession, UCSC RefGene Group; where in respect to gene is CpG located, Beta; estimated coefficient beta from the model, StandardError; standard error, Z; z-score, LRT; likelihood ratio test, Wald; Wald test, LogRank; log-rank test, HR; Hazardous ratio, HR lower; Hazardous ratio lower 95% bound, HR upper; Hazardous ratio upper 95% bound.

**Additional file 10: Table S10.** Description of the eight genes that are predictors of mortality. Data were collected from Gene Ontology (GO) to identify the functional annotation of each gene and recently published COVID-19 related articles to highlight the role of each gene in relation to COVID-19.

**Additional file 11.** Supplementary Figures.

## Data Availability

The data sets generated, used, and analyzed during the current study are available from the corresponding author upon reasonable request. Data will be made available to researchers who provide a methodologically sound proposal to the corresponding author with a signed data access agreement.

## I- Inclusion/Exclusion criteria of participants.

1. For COVID-19 positive patients

## Inclusion criteria:

- 1.Male or female aged over 18 years.
- 2.Tested positive for COVID-19 by Real-Time Quantitative Reverse Transcription.
- 3.Admitted to ICU for critically COVID-19 with acute respiratory distress syndrome (ARDS) under mechanical ventilation.
- 4.Patients satisfying the score of 7 to 9 on the WHO clinical progression scale [1] and severe ARDS according to the Berlin definition [2].

## Exclusion criteria:

- 1.Burn and trauma.
- 2.Any immunological diseases or immunosuppressive medications.
- 3.Other immune-related conditions (cancer, hematological malignancies, bone marrow diseases, or transplant).
- 4.Unsigned informed consent form.
- For non-COVID-19 controls:

## Inclusion criteria:

- 1.Male or female aged over 18 years.
- 2.Signed informed consent form.

## Exclusion criteria:

- 1.Positive COVID-19 infection.
- 2.Person with an infectious syndrome during the last 90 days.
- 3.Person receiving within the last 90 days, a treatment based on: antivirals; antibiotics; antiparasitics; antifungals.
- 4.Person having received within the last 15 days a treatment based on: non-steroidal anti-inflammatory drugs
- 5.Person having received within the last 24 months, a treatment based on: immunosuppressive therapy; corticosteroids; therapeutic antibodies; chemotherapy.
- 6.Person with history of innate or acquired immune deficiency; hematological disease; solid tumor; severe chronic disease; surgery or hospitalization within the last two years; pregnancy within the previous year; participation in a phase I clinical assay in the previous year; participation to a degree I clinical assay in the previous year; pregnant or breastfeeding women; a person with restricted liberty or under legal protection.
- 7.Person with a history of cardiovascular disease.
- 8.Unsigned informed consent form [15, 70]

## Abbreviations

ARDS: Acute respiratory distress syndrome
DMR: Differentially methylated regions
PBMCs: Peripheral blood mononuclear cells
WHO: World health organization
