# Supplementary material for: DNA methylation predicts the outcome of COVID-19 patients with acute respiratory distress syndrome
Source: J Transl Med. 2022 Nov 12;20:526. doi: 10.1186/s12967-022-03737-5 (PMC9652914; doi:10.1186/s12967-022-03737-5)
Supplement: Supplementary file 1 — Additional file 1:Table S1. Baseline characteristics of COVID-19 patients and controls. [file 12967_2022_3737_MOESM1_ESM.docx]

**Supplemental Table 1**. Baseline characteristics of COVID-19 patients and controls

| **Variable** | **COVID-19**  **N = 100^1^** | **Controls**  **N = 33^1^** | **p-value^2^** |
| --- | --- | --- | --- |
| Age | 49 (42, 59) | 40 (35, 45) | <0.001 |
| Gender (male) | 95 (95%) | 33 (100%) | 0.3 |
| BMI (kg/m2) | 27.2 (24.6, 31.0) | 29.3 (26.4, 31.2) | 0.12 |
| Ethnicity |  | | |
| East Africa | 0 (0%) | 1 (3.0%) | <0.001 |
| Middle East | 5 (5.0%) | 11 (33%) |  |
| North Africa | 1 (1.0%) | 0 (0%) |  |
| Northeast Africa | 5 (5.0%) | 4 (12%) |  |
| South Asia | 78 (78%) | 15 (45%) |  |
| Southeast Asia | 9 (9.0%) | 2 (6.1%) |  |
| Western Asia | 2 (2.0%) | 0 (0%) |  |
| Duration of MV (days) | 8 (4, 19) | - |  |
| ICU LoS (days) | 15 (10, 27) | - |  |
| Hospital LoS (days) | 27 (20, 44) | - |  |
| ECMO | 12 (12%) | - |  |
| Nosocomial infections | 55 (55%) | - |  |
| Convalescent plasma therapy | 30 (30%) | - |  |
| *Diabetes status* |  | | |
| Non diabetes | 55 (55%) | - | - |
| Pre-diabetes | 4 (4.0%) | - |  |
| Diabetes | 41 (41%) | - |  |
| Hypertension | 43 (43%) | - | - |
| Coronary artery disease | 6 (6.0%) | - | - |
| Chronic kidney failure | 11 (11%) | - | - |
| Chronic heart failure | 2 (2.0%) | - | - |

*Data are represented as numbers (%) per each category for categorical variables and as median (IQR) for continuous variables. P-values were calculated with Fisher exact test or Wilcoxon rank-sum test. ECMO: extracorporeal membrane oxygenation, LoS= length of stay, MV: mechanical ventilation. ^1^ Median (IQR); n (%), ^2^ Wilcoxon rank-sum test; Fisher's exact test; Pearson's Chi-squared test.*
