# Supplementary material for: DNA methylation predicts the outcome of COVID-19 patients with acute respiratory distress syndrome
Source: J Transl Med. 2022 Nov 12;20:526. doi: 10.1186/s12967-022-03737-5 (PMC9652914; doi:10.1186/s12967-022-03737-5)
Supplement: Supplementary file 4 — Additional file 4: Table S4. Summary of differentially methylated pathways detected between COVID-19 patients and controls based on CpG sites. [file 12967_2022_3737_MOESM4_ESM.docx]

**Supplemental Table 4.** Summary of differentially methylated pathways detected between COVID-19 patients and controls based on CpG sites

| Hallmark pathway | N | DE | P.DE | FDR | Changes in CpGs  (COVID-19 vs. controls) |
| --- | --- | --- | --- | --- | --- |
| Interferon gamma response | 189 | 175 | 0.00 | 0.00 | hypomethylation |
| Estrogen response early | 189 | 174 | 0.00 | 0.02 | hypomethylation |
| Apical surface | 39 | 39 | 0.00 | 0.02 | hypomethylation |
| Interferon alpha response | 93 | 87 | 0.00 | 0.02 | hypomethylation |
| Uv response dn | 140 | 131.33 | 0.00 | 0.02 | hypomethylation |
| Mitotic spindle | 188 | 175 | 0.000 | 0.001 | hypomethylation |

*Columns represent the following variables: N: number of genes in the gene set, DE: number of differentially methylated genes, P.DE: p-value for over-representation of the gene set, FDR: false discovery rate (p-value < 0.05).*
