# Supplementary material for: DNA methylation predicts the outcome of COVID-19 patients with acute respiratory distress syndrome
Source: J Transl Med. 2022 Nov 12;20:526. doi: 10.1186/s12967-022-03737-5 (PMC9652914; doi:10.1186/s12967-022-03737-5)
Supplement: Supplementary file 8 — Additional file 8: Table S8. Description of 27 genes from 49 differentially methylated CpGs between survived and dead patients over four time points. A summarized description of the 27 genes obtained from Supplementary Table 7B, collected from Gene Ontology (GO) to identify the functional annotation of each gene and recently published COVID-19-related articles to highlight the role of each gene in relation to COVID-19. [file 12967_2022_3737_MOESM8_ESM.docx]

**Supplemental table 8:** Description of 27 genes from 49 differentially methylated CpGs between survived and died patients over four time points:

| **RefGene Name** | **CpG position** | **RefGene functional annotation*** | **RefGene molecular process*** | **Associated conditions with RefGene** | **RefGene in COVID-19 related findings** |
| --- | --- | --- | --- | --- | --- |
| DEFB115 | cg00237825 | Protein binding | Innate immune response | Carcinogenesis; Infections | NA |
| DEFB116 | cg13700506 | Protein binding | Innate immune response | Carcinogenesis; Infections | NA |
| SH3PXD2B | cg10547329 | Protein or phosphatidyl-inositol binding;  NADPH superoxidase activity | Eye, heart, bone, adipose tissue development;  Cell differentiation;  Podosome assembly | Frank–Ter Haar syndrome | NA |
| MYOG | cg04395153 | Protein or DNA binding | Myoblast proliferation and differentiation | Rhabdomyosarcoma | NA |
| SOX9-AS1 | cg14668811 | Protein or DNA binding | Osteoblast, myoblast, mesenchymal proliferation;  Immune system inhibition | Cancers | Linked to COVID-19 severity in male (Y-linked) [1] |
| GRIK4 | cg25886627 | Glutamate receptor signaling pathway | NA | Psychiatric disorders | NA |
| ACTL9 | cg20074214 | Protein binding | Acrosome assembly | Infertility; Dermatitis | NA |
| CHST8 | cg10579818 | Protein binding;  Transferase activity | Sulfur, carbohydrate metabolic process;  Center nervous system development | Peeling skin syndrome; Brain metastasis; Encephalopathy | Downregulated in recovered and retested positive COVID-19 patients [2] |
| MAP4K5 | cg20383780 | Protein or ATP binding; Kinase activity | Intracellular signal transduction;  Immune cell infiltration in SONFH° | Osteonecrosis; Cancers | Targeted by SARS-CoV-2 protease (ex-vivo) [3] |
| RPL34 | cg21648245 | RNA binding | Cytoplasmic translation | Cancers | Reported in diabetic gastroenteropathy patients with COVID-19 [4]; Downregulated in COVID-19 patients [5] |
| COL23A1 | cg14863632 | Protein or heparin binding | Extracellular matrix organization | Cancers; Osteoarthritis | Hypermethylated in COVID-19 patients [6]; Interacts with ACE2, TMPRSS2 and FURIN (in-silico) [7] |
| KRT7 | cg22813430 | Protein binding | Composition of ciliated cells in the respiratory mucosa | Cancers; EMT in ovarian cancer | Expressed on ciliated cells, and was insignificant between COVID-19 and control patients [8] |
| MAP2 | cg09475025 | Protein (microtubule; tau) binding | Center nervous system, dendrite development;  Microtubule bundle formation | Multiple neurological disorders | Elevated in COVID-19 patients with a brain injury complication [9] |
| SEC22B | cg06767025 | Protein binding;  SNAP receptor activity | Vesicle-medicated transport (antigens; cytokines; nitric oxide) Autophagosome; assembly negative regulation | Cancers; Neurodegeneration; Infections | NA |
| SPATS2L | cg24419828 | Protein or RNA binding | NA | Cancers; Persistent severe asthma | Overexpressed in COVID-19 patients, limiting viral entry and replication [10] |
| ZDHHC11 | cg20584011 | Protein binding;  Transferase activity | Peptidyl-L-cysteine S-palmitoylation;  Antiviral innate immune response (to DNA viruses) | Cancers | Reported as enhancer for SARS-CoV-2 S-mediated virus entry (ex-vivo) [11] |
| RSPO2 | cg12922129 | Protein or signaling receptor binding | Osteoblast, dopaminergic neuron differentiation;  Epithelial tube branching | Cancers; Tracheomalacia | NA |
| PNMA2 | cg07138115 | Protein binding | Positive regulation of apoptosis | Cancers; Paraneoplastic neurological disorders | Negative in CSF and serum of COVID-19 patients with encephalitis (case reports) [12, 13] |
| TRAF7 | cg03517024 | Protein or ion binding;  Ubiquitin-protein transferase activity | MAPK, ERK1 and ERK2 cascade;  Positive regulation of apoptosis | Cancers; Congenital anomalies | Downregulated in COVID-19 patients [5]; Upregulated in recovered and retested positive COVID-19 patients [2] |
| PTPRN2 | cg11575295 | Phosphatase activity;  Vesicle-mediated secretion activity | Neurotransmitter secretion;  Insulin secretion in cellular response to glucose stimulus | Cancers; Type I diabetes mellitus | Downregulated in COVID-19 patients [5]; Upregulated transcripts in severe COVID-19 patients (in-silico) [14]; Enriched in neutrophil-related pathways in recovered and retested COVID-19 patients [2] |
| SYCE1 | cg20169779 | Protein binding | Meiotic cell cycle: synaptonemal complex assembly | Male infertility; Premature ovarian failure; Cancers | NA |
| MFSD11 | cg07555753 | Protein binding | Ion metabolism and transport | Infections; Cancers Neurological disorders | NA |
| ZNF541 | cg22341310 | Protein or ion binding; transcription corepressor activity | Cell differentiation; Negative regulation of transcription;  Histone deacetylation;  Spermatogenesis | Cancers | NA |
| MTNR1B | cg00528572 | Protein binding;  Melatonin receptor activity | Positive regulation of circadian rhythm; Negative regulation of neuron apoptosis; Insulin secretion;  Vasoconstriction; Cytosolic calcium ion concentration | Type II diabetes mellitus; Cancers | Reported to be essential for melatonin’s effect in SARS-CoV-2 entry (in-silico) [15] |
| PLLP | cg24597989 | Protein binding | Myelination;  Response to wounding | Keratoconus; Neurological disorders; Type II diabetes mellitus; Sarcoidosis | Downregulated in recovered and retested positive COVID-19 patients [2]; Suggested to indirectly enhance viral entry (theoretically) [16] |
| DLGAP3 | cg12679275 | Protein or amyloid-beta binding;  Molecular adaptor activity | Regulation of post-synaptic neurotransmitter receptor activity; Modification of synaptic structure | Neurological disorders | NA |
| CRHBP | cg03286609 | Protein or corticotropin-releasing hormone binding | Cellular response to stimulus (estrogen; xenobiotic; stress; tumor necrosis factor; gonadotropin-releasing hormone; potassium);  Synaptic transmission | Neurological and psychological disorders | Expressed on hematopoietic stem cells which were apoptotic and dysregulated (differentiation) in severe COVID-19 patients [17] |

***** based on GO

° steroid-induced osteonecrosis of the femoral head

**REFERENCES:**

1. Penna, C., et al., *Sex‐related differences in COVID‐19 lethality.* British journal of pharmacology, 2020. **177**(19): p. 4375-4385.

2. Fang, K.-Y., et al., *Screening the Hub Genes and Analyzing the Mechanisms in Discharged COVID-19 Patients Retesting Positive through Bioinformatics Analysis.* 2021.

3. Pablos, I., et al., *Mechanistic insights into COVID-19 by global analysis of the SARS-CoV-2 3CLpro substrate degradome.* Cell reports, 2021. **37**(4): p. 109892.

4. Deb, B., D.R. O’Brien, and A.E. Bharucha, *Duodenal mucosal expression of COVID-19-related genes in health, diabetes gastroenteropathy and functional dyspepsia.* The Journal of Clinical Endocrinology & Metabolism, 2022.

5. Vastrad, B., C. Vastrad, and A. Tengli, *Bioinformatics analyses of significant genes, related pathways, and candidate diagnostic biomarkers and molecular targets in SARS-CoV-2/COVID-19.* Gene Reports, 2020. **21**: p. 100956.

6. Zhou, S., et al., *An epigenome‐wide DNA methylation study of patients with COVID‐19.* Annals of human genetics, 2021. **85**(6): p. 221-234.

7. Hossain, M.S., et al., *Prediction of the Effects of Variants and Differential Expression of Key Host Genes ACE2, TMPRSS2, and FURIN in SARS-CoV-2 Pathogenesis: An In Silico Approach.* Bioinformatics and biology insights, 2021. **15**: p. 11779322211054684.

8. Khan, M., et al., *Visualizing in deceased COVID-19 patients how SARS-CoV-2 attacks the respiratory and olfactory mucosae but spares the olfactory bulb.* Cell, 2021. **184**(24): p. 5932-5949. e15.

9. Savarraj, J., et al., *Brain injury, endothelial injury and inflammatory markers are elevated and express sex-specific alterations after COVID-19.* Journal of neuroinflammation, 2021. **18**(1): p. 1-12.

10. Bui, L.T., et al., *Chronic lung diseases are associated with gene expression programs favoring SARS-CoV-2 entry and severity.* Nature communications, 2021. **12**(1): p. 1-13.

11. Li, D., et al., *Palmitoylation of SARS‐CoV‐2 S protein is critical for S‐mediated syncytia formation and virus entry.* Journal of Medical Virology, 2022. **94**(1): p. 342-348.

12. Fadakar, N., et al., *A first case of acute cerebellitis associated with coronavirus disease (COVID-19): a case report and literature review.* The Cerebellum, 2020. **19**(6): p. 911-914.

13. Salari, M. and M. Etemadifar, *Can COVID‐19 accelerate neurodegeneration?* Clinical Case Reports, 2021. **9**(7).

14. Saheb Sharif-Askari, N., et al., *Enhanced expression of autoantigens during SARS-CoV-2 viral infection.* Frontiers in Immunology, 2021. **12**: p. 2271.

15. Reynolds, J.L. and M.L. Dubocovich, *Melatonin multifaceted pharmacological actions on melatonin receptors converging to abrogate COVID‐19.* Journal of Pineal Research, 2021. **71**(1): p. e12732.

16. Shulgin, A., et al., *Plasmolipin and Its Role in Cell Processes.* Molecular Biology, 2021. **55**(6): p. 773-785.

17. Wang, X., et al., *Dysregulated hematopoiesis in bone marrow marks severe COVID-19.* Cell discovery, 2021. **7**(1): p. 1-18.
