# Supplementary material for: DNA methylation predicts the outcome of COVID-19 patients with acute respiratory distress syndrome
Source: J Transl Med. 2022 Nov 12;20:526. doi: 10.1186/s12967-022-03737-5 (PMC9652914; doi:10.1186/s12967-022-03737-5)
Supplement: Supplementary file 10 — Additional file 10: Table S10. Description of the eight genes that are predictors of mortality. Data were collected from Gene Ontology (GO) to identify the functional annotation of each gene and recently published COVID-19 related articles to highlight the role of each gene in relation to COVID-19. [file 12967_2022_3737_MOESM10_ESM.docx]

**Supplemental Table 10:** Description of the eight genes that are predictors of mortality

| **RefGene Name** | **CpG position** | **RefGene functional annotation*** | **RefGene molecular process*** | **RefGene in COVID-19 related findings** |
| --- | --- | --- | --- | --- |
| H1F0 | cg27424226 | Nucleosomal DNA binding;  RNA binding; protein binding | Negative regulation of DNA recombination and chromatin silencing; Nucleosome positioning | Low affinity to COVID-19 protein (Nsp3) in different cell lines ex-vivo [1] |
| ROCK1 | cg26121915 | Nucleotide binding; Tau protein binding; Transferase or kinase activity | Epithelial to mesenchymal transition;  Negative regulation of angiogenesis;  Apoptotic process; others | Proposed to be targeted to limit the inflammation leading to ARDS associated with COVID-19 [2] |
| ZNF789 | cg05563613 | DNA or metal ion binding; Transcription factor activity | Regulation of transcription; RNA metabolic process | NA |
| MAT2B | cg14447399 | Protein binding; Methionine adenosyl-transferase regulator activity | S-adenosylmethionine biosynthetic process; Regulation of catalytic activity | Significantly higher in the enriched cells compared to depleted cells, suggested as a potential contributing gene to genetic risk for severe COVID-19 [3] |
| YY1P2 | cg12406406 | Transcription factor activity | YY1: Spermatogenesis;  B cell differentiation; Cellular response to UV or DNA damage or IL-1 | NA |
| MFHAS1 | cg01784220 | Protein (ligase, phosphatase) binding;  Nucleotide binding | Innate immune response (inflammation, TLR signaling, regulation of macrophage activation); Positive regulation of ERK1 and ERK2 cascade | Enriched in pro-inflammatory pathways and highly expressed in non-vaccinated vs vaccinated non-human primates with COVID-19 [4] |
| MRPS2 | cg12438037 | Ribosome subunit construction | Mitochondrial ribosome assembly and translation | Interacts with COVID-19 protein (Nsp8) [5]; Linked to viral replication and translation in infected cells [6]; Upregulated in COVID-19 recovered and retested positive patients [7] |
| PSMB9 | cg19760441 | Protein binding; peptidase or hydrolase activity | Immune system process; Catabolic process;  Regulation of mRNA stability; Post-translation modification | Significantly expressed in COVID-19 patients with low compared to high viral load in the lung, and in macrophages of mild vs. severe cases [8]; Enriched in cytokine-mediated signaling pathway from upregulated genes in infected tissues [9] |

***** based on GO

**References:**

1. Wang, J.Y., et al., *A master autoantigen-ome links alternative splicing, female predilection, and COVID-19 to autoimmune diseases.* Journal of Translational Autoimmunity, 2022: p. 100147.

2. Bonnet, R., L. Mariault, and J.-F. Peyron, *Identification of potentially anti-COVID-19 active drugs using the connectivity MAP.* PloS one, 2022. **17**(1): p. e0262751.

3. Yu, F., et al., *Variant to function mapping at single-cell resolution through network propagation.* bioRxiv, 2022.

4. Furuyama, W., et al., *Rapid protection from COVID-19 in nonhuman primates vaccinated intramuscularly but not intranasally with a single dose of a recombinant vaccine.* bioRxiv, 2021.

5. Gordon, D., et al., *A SARS-CoV-2-human protein-protein interaction map reveals drug targets and potential drug-repurposing. bioRxiv.* Preprint posted March, 2020. **22**.

6. Chen, F., et al., *A systems‐level study reveals host‐targeted repurposable drugs against SARS‐CoV‐2 infection.* Molecular systems biology, 2021. **17**(8): p. e10239.

7. Fang, K.-Y., et al., *Screening the Hub Genes and Analyzing the Mechanisms in Discharged COVID-19 Patients Retesting Positive through Bioinformatics Analysis.* 2021.

8. Desterke, C., et al., *HLA-dependent heterogeneity and macrophage immunoproteasome activation during lung COVID-19 disease.* Journal of translational medicine, 2021. **19**(1): p. 1-19.

9. Elkahloun, A.G. and J.M. Saavedra, *Candesartan could ameliorate the COVID-19 cytokine storm.* Biomedicine & Pharmacotherapy, 2020. **131**: p. 110653.
