# Supplementary material for: DNA methylation predicts the outcome of COVID-19 patients with acute respiratory distress syndrome
Source: J Transl Med. 2022 Nov 12;20:526. doi: 10.1186/s12967-022-03737-5 (PMC9652914; doi:10.1186/s12967-022-03737-5)
Supplement: Supplementary file 11 — Additional file 11. Supplementary Figures. [file 12967_2022_3737_MOESM11_ESM.pdf]

Supplemental Figures legends

Supplemental Figure 1. PCA plot of controls and COVID-19 patients at baseline for 750874 CpG sites. Representation by COVID-19 (red dots) and controls (blue dots) is shown, and the proportion of variance is explained by each PC (PC1 and PC2).

Supplemental Figure 2. Representation of the Akaike Information Criterion (AIC) for the comparison of different linear models tested to determine the best model suitable for the analysis. Every letter corresponds to a different tested model: **(A)** None=no covariates. A= Age. B=Ethnicity. C=Age, ethnicity. D=Age, ethnicity, the proportion of CD8 T cells, CD4 T cells, and B cells. E=Age, the proportion of CD8 T cells, CD4 T cells, and B cells. F=The proportion of CD8 T cells, CD4 T cells, and B cells. G= The proportion of CD8 T cells and CD4 T cells. H= The proportion of CD8 T cells. I= The proportion of B cells. J= The proportion of CD4 T cells. **(B).** Different letters correspond to a different tested model: None= CpG methylation ~ Dead/recovered, A= CpG methylation ~ Dead/recovered + Age, B= CpG methylation ~ Dead/recovered + Age + MV days, C= CpG methylation ~ Dead/recovered + Age + MV days + Gender, D= CpG methylation ~ Dead/recovered + Age + MV days + Gender + ICU stay, E= CpG methylation ~ Dead/recovered + Age + MV days + Gender + ICU LoS + ECMO, J= CpG methylation ~ Dead/recovered + Age + MV days + Gender + ICU LoS + ECMO + Nosocomial infections, K= CpG methylation ~ Dead/recovered + Age + MV days + ICU LoS + ECMO, L= CpG methylation ~ Dead/recovered + ICU LoS + ECMO, M= CpG methylation ~ Dead/recovered + Age + ICU LoS, G= CpG methylation ~ Dead/recovered + Age + ICU stay + ECMO, H= CpG methylation ~ Dead/recovered + Age + MV days + ICU LoS + ECMO + Nosocomial infections, I= CpG methylation ~ Dead/recovered + Age + MV days + Gender + ECMO.

Supplemental Figure E3: Miami plot from the genome-wide differential methylation between COVID-19 and controls. The plot shows the result of the COVID-19 severity association test over 750874 CpG positions. The red line designates the genome-wide significance threshold of a Benjamini-Hochberg corrected P-value < 0.05. Green dots represent significantly different CpGs from the previously reported genes associated with COVID-19[1-7]. The upper panel represents hypermethylated CpGs, while the lower panel represents hypomethylated CpGs.

1. Castro de Moura M, Davalos V, Planas-Serra L, Alvarez-Errico D, Arribas C, Ruiz M, et al. Epigenome-wide association study of COVID-19 severity with respiratory failure. EBioMedicine. 2021;66:103339.
2. Severe Covid GG, Ellinghaus D, Degenhardt F, Bujanda L, Buti M, Albillos A, et al. Genomewide Association Study of Severe Covid-19 with Respiratory Failure. N Engl J Med. 2020;383(16):1522-34.
3. Pairo-Castineira E, Clohisey S, Klaric L, Bretherick AD, Rawlik K, Pasko D, et al. Genetic mechanisms of critical illness in COVID-19. Nature. 2021;591(7848):92-8.
4. Zhang Q, Bastard P, Liu Z, Le Pen J, Moncada-Velez M, Chen J, et al. Inborn errors of type I IFN immunity in patients with life-threatening COVID-19. Science. 2020;370(6515).
5. Bastard P, Rosen LB, Zhang Q, Michailidis E, Hoffmann HH, Zhang Y, et al. Autoantibodies against type I IFNs in patients with life-threatening COVID-19. Science. 2020;370(6515).
6. van der Made CI, Simons A, Schuurs-Hoeijmakers J, van den Heuvel G, Mantere T, Kersten S, et al. Presence of Genetic Variants Among Young Men With Severe COVID-19. Jama-J Am Med Assoc. 2020;324(7):663-73.
7. Singh H, Choudhari R, Nema V, Khan AA. ACE2 and TMPRSS2 polymorphisms in various diseases with special reference to its impact on COVID-19 disease. Microb Pathogenesis. 2021;150.

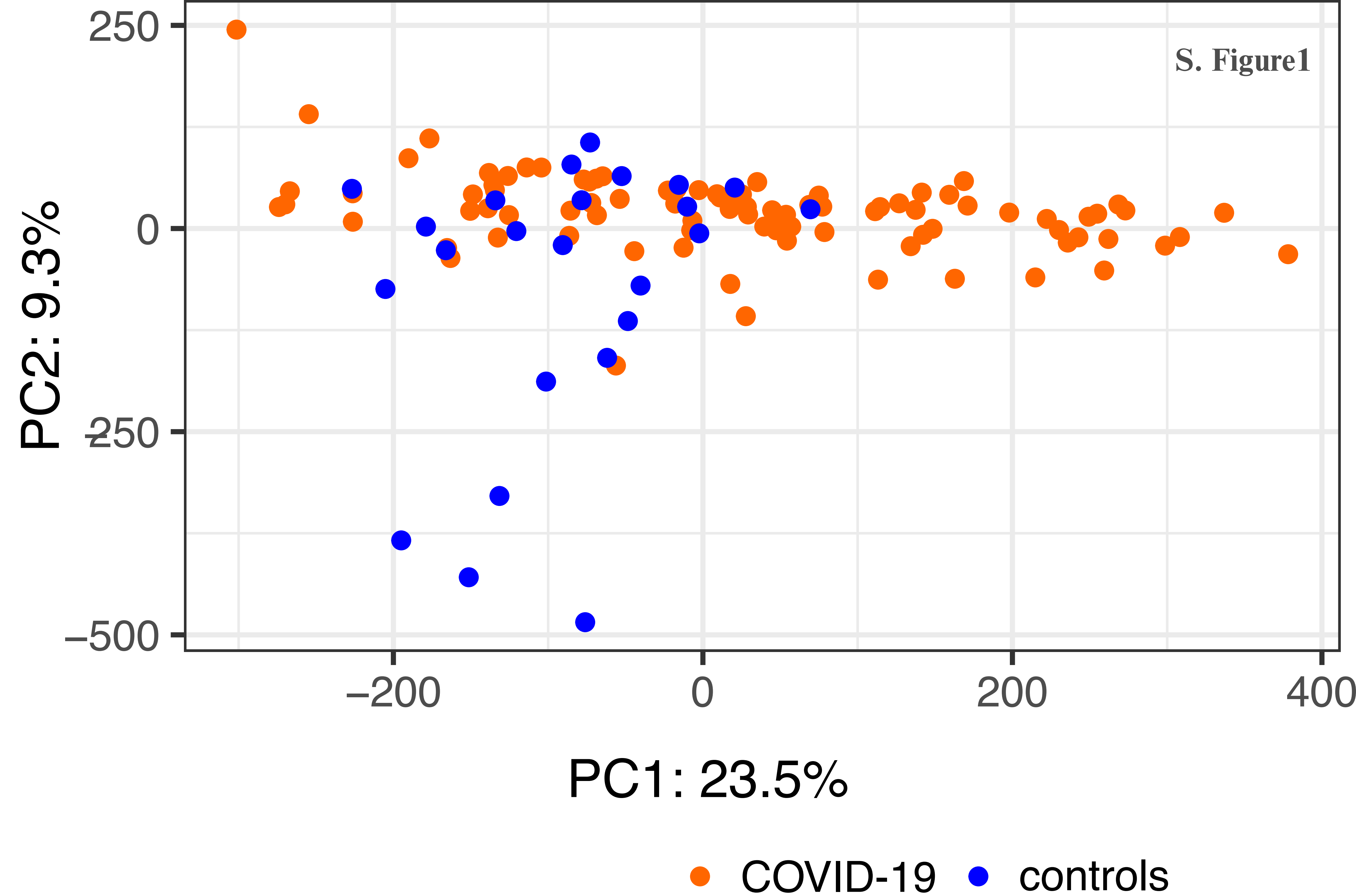

S. Figure1
250
0
-250
-500
PC2: 9.3%
-200
0
200
400
PC1: 23.5%
COVID-19
controls

S. Figure 2

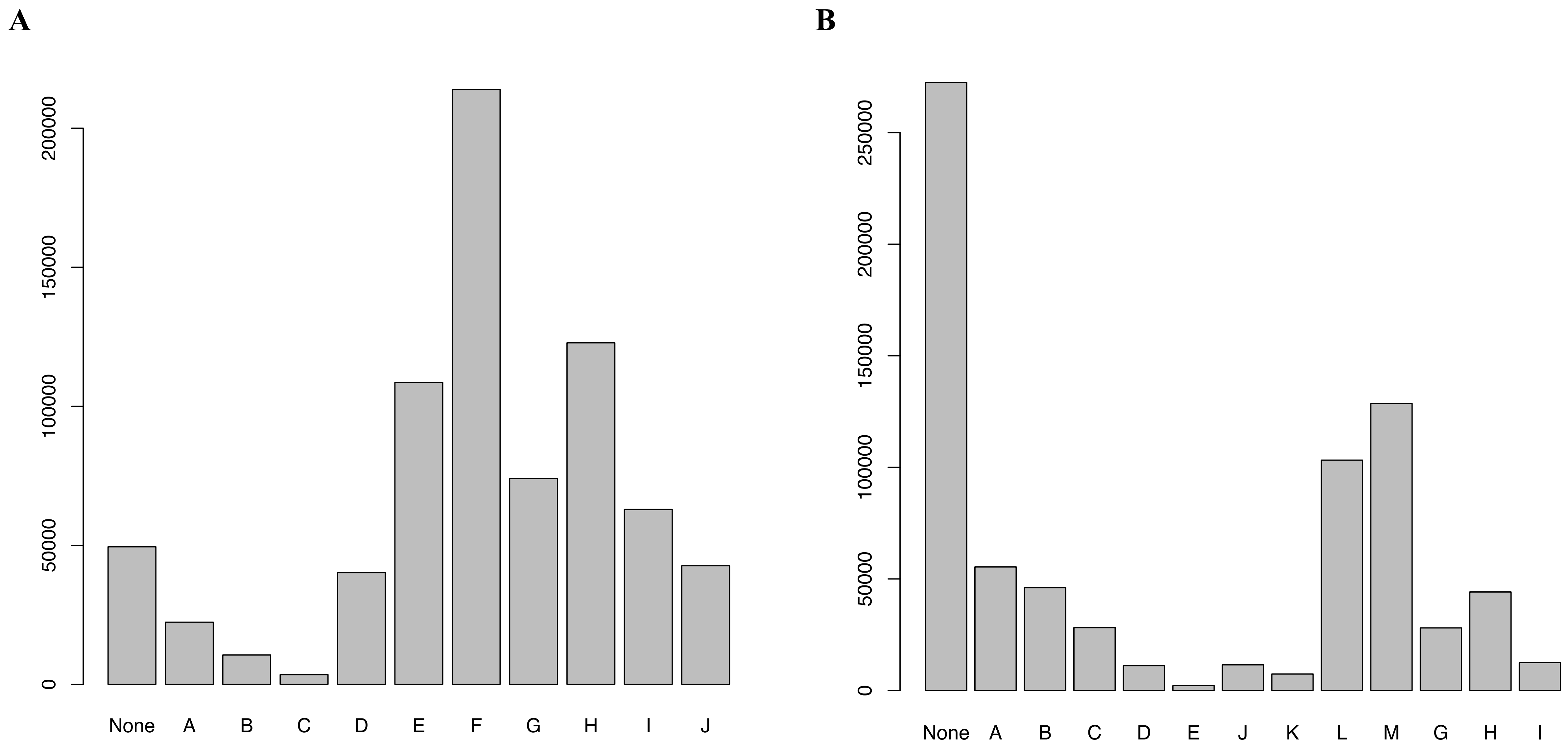

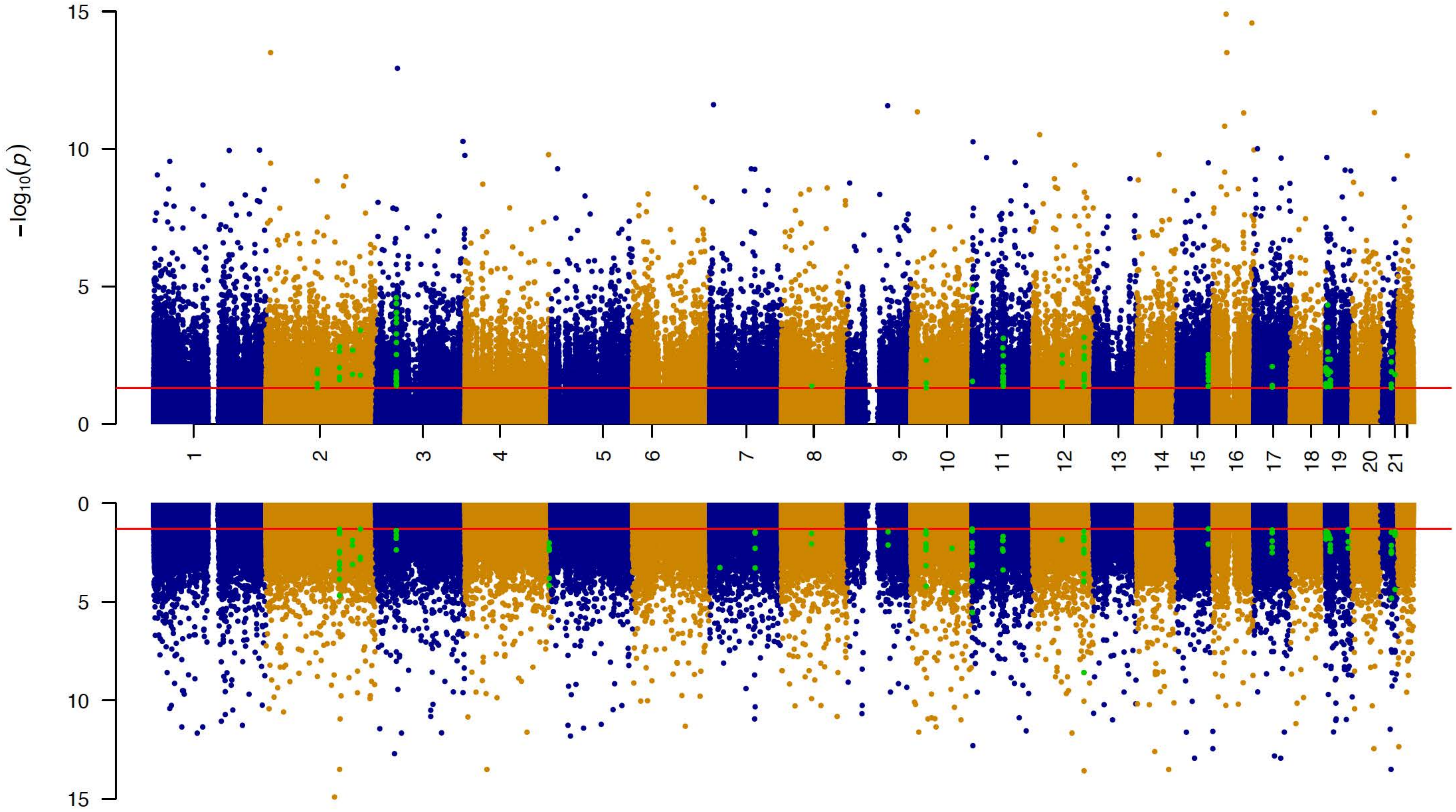
15
10
5
0
-log10(p)
1
2
3
4
5
6
7
8
9
10
11
12
13
14
15
16
17
18
19
20
21
0
5
10
15
